# A Specific tRNA Half, 3'tiRNA‐GlyGCC, Regulates Hypoxic Pulmonary Artery Smooth Muscle Cell Proliferation via Myrf‐Mediated Endoplasmic Reticulum Stress

**DOI:** 10.1111/cpr.70238

**Published:** 2026-05-29

**Authors:** Lixin Zhang, Xiaoyu Guan, Xiangrui Zhu, Yingying Hao, Ya Xu, Hao Yuan, Danni Gao, Hongxia Du, Jiayu Li, Weiwei Cao, Cui Ma, Xiaoying Wang

**Affiliations:** ^1^ College of Medical Laboratory Science and Technology Harbin Medical University, Daqing Campus Daqing P. R. China; ^2^ Central Laboratory of Harbin Medical University, Daqing Campus Daqing P. R. China; ^3^ College of Pharmacy, Harbin Medical University Harbin P. R. China; ^4^ Institute of Cardiovascular Diseases, Xiamen Cardiovascular Hospital, School of Medicine, Xiamen University, Fujian Branch of National Clinical Research Center for Cardiovascular Diseases Xiamen P. R. China; ^5^ College of Pharmacy, Harbin Medical University, Daqing Campus Daqing P. R. China; ^6^ Key Laboratory of Frigid Zone Exercise Health Research and Translation in Heilongjiang Province Daqing P. R. China

**Keywords:** 3′tiRNA‐GlyGCC, endoplasmic reticulum stress, proliferation, pulmonary artery smooth muscle cell, pulmonary hypertension

## Abstract

Pulmonary artery smooth muscle cell (PASMC) proliferation is a hallmark of the pathogenesis of hypoxic pulmonary hypertension (PH), and endoplasmic reticulum stress (ERS) plays a crucial role. Many studies have implicated that tRNA‐derived fragment, tiRNAs, in a variety of biological processes, but their roles in hypoxia‐induced PASMC ERS and proliferation have not been investigated. In this study, we identified a significantly upregulated 3′tiRNA‐GlyGCC in hypoxic mouse lung tissues using Arraystar small RNA microarray analysis. Functional assays, including CCK8, EdU incorporation, Western blot, and immunofluorescence, demonstrated that inhibition of 3′tiRNA‐GlyGCC reversed hypoxia‐induced ERS and proliferation in PASMCs. Mechanistically, 3′tiRNA‐GlyGCC interacts with the eukaryotic translation elongation factor 1 alpha 1 (Eef1a1) protein and reduces the binding capacity between Eef1a1 and myelin regulatory factor (Myrf) mRNA, leading to decreased stability of Myrf mRNA. Additionally, 3′tiRNA‐GlyGCC targets Myrf mRNA and inhibits its expression. We further verified that angiogenin (Ang) mediated the biogenesis of 3′tiRNA‐GlyGCC under hypoxic conditions. Collectively, these findings highlight a novel mechanism underlying PASMC ERS and proliferation and suggest that 3′tiRNA‐GlyGCC could serve as a potential therapeutic target for hypoxic PH.

## Graphical abstract

A schematic diagram illustrating how 3′tiRNA‐GlyGCC promotes the endoplasmic reticulum stress (ERS) and proliferation in hypoxic pulmonary artery smooth muscle cells (PASMCs) by inhibiting the expression of myelin regulatory factor (Myrf), ultimately leading to pulmonary hypertension (PH).

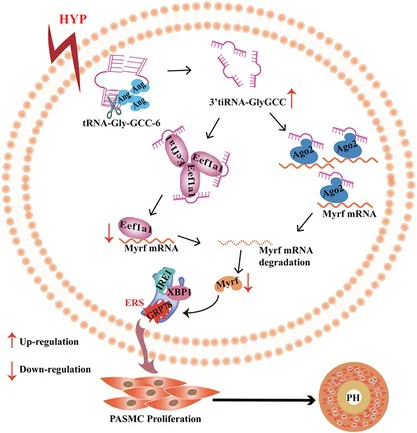

**Abbreviations**
3'UTR: 3' untranslated region
AAV9: Serotype 9 adenovirus‐associated virus
Eef1a1: eukaryotic translation elongation factor 1 alpha 1
ERS: endoplasmic reticulum stress
FISH: fluorescence in situ hybridization
LVEF: left ventricular ejection fraction
Myrf: myelin regulatory factor
PAAT: pulmonary artery acceleration time
PASMC: pulmonary artery smooth muscle cell
PAVTI: pulmonary artery velocity time integral
PH: pulmonary hypertension
RVSP: right ventricular systolic pressure
tsRNAs: tRNA‐derived fragments
WT: wild‐type

## 1 Background

Pulmonary hypertension (PH) is one of the most common cardiovascular and respiratory system diseases and has a high mortality rate. Abnormal vascular remodelling, which results in right ventricular hypertrophy and death, is a key feature of pulmonary vasculature pathological changes in PH [1, 2]. Pulmonary vascular remodelling involves various cellular pathological factors, such as cell proliferation, pyroptosis, autophagy, and calcification [3, 4, 5, 6, 7, 8, 9]. The hyperproliferation of pulmonary artery smooth muscle cells (PASMCs) constitutes the primary driver of pulmonary vascular remodelling. However, elucidating the precise molecular and cellular mechanisms underlying PASMC proliferation remains challenging. Therefore, thoroughly investigating the pathological mechanisms that drive PASMC proliferation is crucial for identifying novel therapeutic targets for the treatment of PH.

Endoplasmic reticulum stress (ERS) refers to the accumulation of misfolded or unfolded proteins in the endoplasmic reticulum caused by some exogenous or endogenous factors, thereby damaging the normal physiological functions of the endoplasmic reticulum [10]. ERS is mainly mediated by a set of stress sensors including IRE1, PERK, and ATF6, which are located on the ER membrane [11]. Under stress conditions, these factors directly activate the transcription of molecular chaperones or proteins and regulate various diseases, such as cancer, cardiovascular diseases, liver diseases, and diabetes, through various cellular processes [12, 13, 14, 15]. Accumulating evidence has shown that multiple signalling molecules and signal transduction pathways are involved in the regulatory effect of ERS on PASMC proliferation. For example, lipocalin‐2 (Lcn2) can promote PASMC ERS and proliferation by augmenting the level of intracellular iron [16]. Neurotensin receptor 1 (Ntsr1) can induce enhanced ERS and increase PASMC proliferation and migration via the JAK2‐STAT3‐Thbs1 signalling pathway [17]. Given that the molecular mechanisms underlying ERS regulation in PH are relatively complex, they remain unclear. Therefore, identifying additional regulators of ERS in PH is crucial.

For decades, noncoding RNAs (ncRNAs), including microRNAs (miRNAs), long noncoding RNAs (lncRNAs), piwi‐interacting RNAs (piRNAs), and circular RNAs (circRNAs), have been identified as important regulators involved in the pathological process of many diseases. Intriguingly, recent studies have revealed tRNA‐derived fragments (tsRNAs) to be a hot topic in the biomedical field. tsRNAs are classified into two major types: tRNA halves (tiRNAs, 30–45 nucleotides) and smaller tRNA fragments (tRFs, 14–30 nucleotides). Many reports have shown that tsRNAs play important roles in many diseases by regulating cell proliferation, the inflammatory response, and DNA damage. Xiong et al. reported high expression of tiRNA‐Val‐CAC‐2 in pancreatic cancer tissue and found a positive correlation between the content of tiRNA‐Val‐CAC‐2 and tumour metastasis [18]. Cai et al. showed that Ang‐induced 5′‐tsRNAs inhibited NLRP3 inflammasome activation and pyroptosis, making them promising targets for the treatment of inflammasome‐related diseases [19]. In addition, a recent study revealed that tRF‐1‐AspGTC regulates hypoxia‐induced PASMC proliferation and apoptosis resistance in PH, suggesting that it may be a potential diagnostic biomarker for PH [20]. Therefore, identifying and characterising such tsRNAs as therapeutic targets in the pathogenesis of PH is highly important.

The present study explored several new functional tiRNAs and clarified their roles in pulmonary vascular remodelling. Notably, 3′tiRNA‐GlyGCC was significantly upregulated in hypoxic lung tissues and PASMCs. We further investigated the role and mechanism of 3′tiRNA‐GlyGCC in ERS and proliferation of PASMCs under hypoxia. Our data uncover a critical function of tiRNAs in the regulation of pulmonary vascular remodelling, providing a new therapeutic target for pharmaceutical intervention in PH.

## 2 Methods

### 2.1 Animal Experimental Protocols

To avoid the influence of estrogen on SU5416 combined with hypoxia (SuHx)‐induced PH, healthy male C57BL/6J mice (6 weeks old, weighing 20–25 g each) were used in this study [21, 22]. All animals were obtained from the Laboratory Animal Center of the Second Affiliated Hospital of Harbin Medical University and were randomly and evenly divided into the serotype 9 adenovirus‐associated virus (AAV9)‐negative control (NC) group, AAV9‐3′tiRNA‐GlyGCC inhibitor group, SuHx+AAV9‐NC group, and SuHx+AAV9‐3′tiRNA‐GlyGCC inhibitor group. The corresponding target RNA cloning construct and AAV9 were packaged by Genechem (Shanghai, China). Approximately 1 × 10^11^ genome equivalent vectors were prepared in Hank's balanced salt solution (HBSS) with 20–30 μL, and the mice were infected via nasal drops. The infected mice were randomly divided into two groups: the normal oxygen group (Fi, O_2_ 0.21) and the hypoxia (Fi, O_2_ 0.10) group. The normal oxygen group was maintained at the same location adjacent to the hypoxic group, and the concentration of oxygen was monitored continuously via an oxygen analyser (P110, BioSpherix New York) for 4 weeks. For the SuHx model, the mice were injected subcutaneously with a single dose of the VEGFR inhibitor SU5416 (20 mg/kg, Sigma‐Aldrich, USA) for the next 3 weeks, whereas the control mice received vehicle injections.

### 2.2 Echocardiography Measurement

Echocardiography was performed with a Vevo2100 imaging system (VisualSonics, Toronto, Ontario, Canada). Briefly, the mice were anaesthetised through an anaesthetic mask containing isoflurane (concentration: 1%–2%; flow rate: 0.6–1 L/min, R510‐22, RWD, Shenzhen, China) and secured on a prewarmed (37°C) imaging platform. The pulmonary artery velocity time integral (PAVTI), pulmonary artery acceleration time (PAAT), left ventricular ejection fraction (LVEF), and heart rate were obtained after the fur on the chest of each mouse was removed. To ensure consistency and accuracy, echocardiography measurements and offline data analysis were performed by experienced investigators in a blinded manner.

### 2.3 Right Ventricular Systolic Pressure (RVSP) and Right Ventricular Hypertrophy Assay

The right ventricular systolic pressure (RVSP) was measured with PowerLab monitoring equipment (AD Instruments, Colorado Springs, CO). Briefly, the mice were anaesthetised through an intraperitoneal injection of avertin (200 mg/kg, Sigma Aldrich, St. Louis, MO), and a 1.2 French pressure catheter (Scisense Inc., London, Ontario, Canada) was inserted into the superior vena cava and ultimately into the right ventricular vein. After the RVSP was recorded, the heart and lung tissues of the mice were rapidly excised via surgical scissors. The right ventricular hypertrophy index (the ratio of the RV free wall weight over the sum of the septum plus left ventricular (LV) free wall weight), RV/(LV + S), was calculated. To ensure consistency and accuracy, all measurements and offline data analyses were performed by experienced investigators in a blinded manner.

### 2.4 Morphometric Analysis

Fresh tissue was fixed with 4% paraformaldehyde for more than 24 h and embedded in paraffin. The wax blocks were cut into 5 μm thick slices for haematoxylin–eosin (HE) staining. The paraffin‐embedded sections were deparaffinised, stained with haematoxylin, dehydrated, permeabilized, sealed with neutral gum, and dried. Images were taken using an Aperio VERSA 8 Scanning System (Leica, Teaneck, NJ) and then analysed.

### 2.5 Arraystar Small RNA Microarray Analysis

Arraystar mouse small RNA modification microarray analysis was performed via Aksomics (H2108075A, Shanghai). In brief, RNA quantity was measured via a NanoDrop ND‐1000 spectrophotometer and RNA integrity was assessed by Bioanalyzer 2100 or gel electrophoresis. For each sample, 100 ng of total RNA was first dephosphorylated to form the 3‐OH end. The 3‐OH containing RNA was then denatured with DMSO and enzymatically labelled with Cy3. The labelled RNA was hybridized onto an Arraystar Mouse small RNA Microarray (8 × 15K, Arraystar) and the array was scanned with an Agilent Scanner G2505C. Quantile normalisation and subsequent data processing were performed via the GeneSpring GX v12.1 software package (Agilent Technologies). After normalisation, the probe signals with Present (P) or Marginal (M) QC flags in at least 5 out of 10 samples were retained. Differentially expressed tsRNAs (tRFs and tiRNAs) between the two comparison groups were identified by fold change (FC) and statistically significant (*p*‐value) thresholds.

### 2.6 Cell Culture

Mouse pulmonary arterial smooth muscle cells (PASMCs) were obtained from Procell Life Science and Technology (Wuhan, China), and passage 3–7 PASMCs were used. The cells were maintained in Dulbecco's modified Eagle's medium (DMEM) complete medium (KeyGEN, Nanjing, China), containing 15% fetal bovine serum (FBS, Clark, USA) and 1% penicillin–streptomycin at 37°C, 5% CO_2_, and 100% relative humidity. To induce hypoxia, the cells were placed in a Tri‐Gas Incubator (Thermo, Waltham, MA) in a water‐saturated atmosphere containing 3% O_2_, 5% CO_2_, and 92% N_2_ for 24 h.

### 2.7 Western Blot Analysis

Total protein was extracted from PASMCs via RIPA lysis buffer (P0013B; Beyotime Biotechnology, Shanghai, China) supplemented with phenylmethylsulfonyl fluoride (ST506; Beyotime Biotechnology, China). The proteins were separated via 10% or 12% SDS–PAGE and transferred to a polyvinylidene fluoride (PVDF) membrane. The membranes were subsequently blocked in 5% nonfat dry milk for 1 h. The primary antibody was incubated with the membrane overnight at 4°C, followed by incubation with horseradish peroxidase‐labelled secondary antibodies at room temperature. The membranes were subsequently subjected to chemiluminescence reagent imaging and quantified via Quantity One software (Bio‐Rad Laboratories, Hercules, CA). The following primary antibodies were used: PCNA (1:1000, 10205‐2‐AP, Proteintech, Wuhan, China), Cyclin D (1:500, BM4272, BOSTER, Wuhan, China), Cyclin A (1:1000, 82148‐1‐RR, Proteintech, Wuhan, China), IRE1 (1:1000, # 3294, Cell signalling, MA, USA), XBP1 (1:500, A1731, ABclonal, Wuhan, China), GRP78 (1:2000, 11587‐1‐AP, Wuhan, China), Eef1a1 (1:1000, 11402‐1‐AP, Wuhan, China), Myrf (1:500, A16355, ABclonal, China), ATF6 (1:500, A27877, ABclonal, China), CHOP (1:500, A00311, Boster, Wuhan, China), and Ang (A00146, Boster, Wuhan, China).

### 2.8 RNA Isolation and Quantitative Real‐Time Polymerase Chain Reaction

Total RNA was extracted from cells and lung tissues via Trizol reagent (Thermo Fisher, MA) according to the manufacturer's protocol. Then, the mRNA was converted to cDNA using Superscript First‐Strand cDNA Synthesis Kit (HaiGene, Harbin, China), and the tsRNAs were reverse‐transcribed with a specific primer (GenePharma, Suzhou, China). Finally, a real‐time PCR system (Roche (Basel, Switzerland) or TIANLONG (Xi'an, China)) with SYBR Green (TOYOBO, Osaka, Japan) was used to conduct RT–qPCR. Samples were normalized to β‐actin or U6 expression via the 2^−ΔΔCT^ method. The primers (5′‐3′) used are shown in Supplementary file: Table S1.

### 2.9 Immunofluorescence Staining Analysis

The treated PASMCs were fixed with 4% paraformaldehyde and permeabilized with 0.3% Triton X‐100. After being blocked with 5% normal bovine serum, the PASMCs were incubated at 4°C overnight with primary antibodies against GRP78 (1:100, 11587‐1‐AP, Wuhan, China), Eef1a1 (1:100, 11402‐1‐AP, Wuhan, China), and Myrf (1:50, CSB‐PA897527LA01HU, CUSABIO, Wuhan, China). The cells were subsequently incubated with Cy3/FITC conjugated secondary antibody (1:100) at 37°C for 2 h and then with DAPI in the dark for 10 min. For tissue samples, lung sections were incubated with GRP78 (1:100, 11587‐1‐AP, Wuhan, China), α‐smooth muscle actin antibody (1:100, #19245; Cell Signalling Technology), and Myrf (1:50, CSB‐PA897527LA01HU, CUSABIO, Wuhan, China). Approximately 5 arteries per animal were randomly examined under a live cell workstation (Leica, Wetzlar, Germany) or confocal microscope system (Nikon, Japan).

### 2.10 3′tiRNA‐GlyGCC Mimics, Inhibitor, and Small Interfering RNA (siRNA) Construction and Transfection

PASMCs were cultured in the well plates overnight before transfection. The 3′tiRNA‐GlyGCC mimics, 3′tiRNA‐GlyGCC inhibitor, siRNA against Myrf, Eef1f1, Ang, and negative control (NC) were designed and synthesised by JTS Scientific (Wuhan, China). X‐tremeGene siRNA reagent was used as the transfection aid reagent according to the manufacturer's protocol. The sequences are shown in Supplementary file: Table S2.

### 2.11 Fluorescence In Situ Hybridization (FISH)

FISH assays were conducted to detect the localisation of 3′tiRNA‐GlyGCC in PASMCs and tissues using a Fluorescent In Situ Hybridization Kit (GenePharma, Shanghai, China) following the manufacturer's instructions. Briefly, the treated PASMCs were fixed with 4% paraformaldehyde and permeabilized with 0.3% Triton X‐100. Subsequently, Cy3‐labelled 3′tiRNA‐GlyGCC, FITC‐labelled 3′UTR of Myrf mRNA, U6, and 18S probes were hybridized overnight at 37°C. For tissue samples, lung tissue sections were pretreated in PBS and incubated overnight with Cy3‐labelled 3′tiRNA‐GlyGCC probe in hybridization buffer at 37°C. Nuclei were stained with DAPI reagent. Finally, images were recorded using a live cell workstation (Leica, Wetzlar, Germany) or confocal microscope system (Nikon, Japan). The probe sequences are shown in Supplementary file: Table S3.

### 2.12 CCK8 Assay

Approximately 5000 cells/well were cultured in 96‐well plates. Following cell transfection for 24 h, 10 μL of Cell‐Counting Kit‐8 reagent (Biosharp, Hefei, China) was added to each well and incubated at 37°C for 2 h. Finally, the results were detected at a wavelength of 450 nm via a spectrophotometer microplate reader.

### 2.13 5‐Ethynyl‐2′‐Deoxyuridine (EdU) Staining

Cell proliferation was detected using a BeyoClick EdU Cell Proliferation Kit (C0075S; Beyotime, Shanghai, China). Briefly, the treated PASMCs were incubated with 50 μmol/L EdU solution at 37°C for 2 h, fixed with 4% paraformaldehyde, and exposed to 0.5% Triton X‐100. Finally, the cells were observed and imaged using a live cell workstation (AF6000; Leica).

### 2.14 RNA Pull‐Down Assay

The RNA‐pulldown assay was performed using the BersinBioTM RNA pull‐down Kit (Bes5102, Guangzhou, China) according to the manufacturer's protocol. A Biotin‐labelled 3′tiRNA‐GlyGCC probe was designed and synthesised by JTS Scientific (Wuhan, China). The collected protein samples bound to RNA were used for mass spectrometry and western blot analysis.

### 2.15 mRNA‐Seq Analysis

The mRNA‐seq analysis was performed by Guangzhou Epibiotek Co Ltd. In brief, PASMCs were transfected with the 3′tiRNA‐GlyGCC inhibitor or NC under hypoxic conditions. Total RNAs were isolated using Trizol reagent (Thermo Fisher, MA). A Neb Next Ultra RNA Library Prep Kit for Illumina (Neb, Ipswich, MA, USA) was used to generate the sequencing libraries. Reads were aligned with Hisat2 to the mouse genome (Ensemble Mouse GRCm38).

### 2.16 RNA Immunoprecipitation (RIP) Assay

The RNA immunoprecipitation (RIP) assay was performed using the BersinBioTM RIP Kit (Bes5101, Guangzhou, China) according to the manufacturer's protocol. Briefly, the PASMC samples were incubated with protein A/G bead–conjugated anti‐Eef1a1 (5 μg; 11402‐1‐AP, Proteintech, Wuhan, China), anti‐AGO2 (5 μg; ab186733, Abcam, USA) antibodies, or IgG overnight at 4°C. RT–qPCR was performed to detect the expression of 3′tiRNA‐GlyGCC and Myrf mRNA.

### 2.17 Dual‐Luciferase Reporter Assay

A dual‐luciferase reporter assay was used to evaluate the direct binding between the 3'UTR regions of Myrf and 3′tiRNA‐GlyGCC. The 3'UTR regions of the Myrf wild‐type (Myrf‐WT) and mutant (Myrf‐MUT) luciferase reporter plasmids were designed and synthesised by JTS Scientific (Wuhan, China). 293 T cells were cotransfected with the 3′tiRNA‐GlyGCC mimic and the mRNA of the Myrf plasmid expressing luciferase for 48 h. Then, the assay was performed according to the protocol of the Dual‐Luciferase Reporter Gene Assay Kit (RG027, Beyotime, China), and the luciferase activities were detected via the dual‐luciferase reporter assay system (GloMax Multi Detection System, Promega, Madison).

### 2.18 Statistical Analysis

All the statistical analyses were carried out using GraphPad Prism 8.0 software (GraphPad Software Inc., La Jolla, CA, USA). The data were checked for normal distribution (Shapiro–Wilk test) and equal variance (Brown–Forsythe test) before statistical testing. Student's *t*‐test was used to compare the data between 2 groups with equal variance, and the Welch correction test was used for 2‐group analysis with unequal variance. One‐way ANOVA with Tukey's post hoc test was used to compare multiple groups with equal variance, and Brown–Forsythe and Welch ANOVA with Tamhane T2 post hoc tests were used to compare multiple groups with unequal variance. For nonnormally distributed data, we performed nonparametric analyses such as the Mann–Whitney *U* test for 2 groups or the Kruskal–Wallis test followed by the Dunn posttest for multiple groups. The results are presented as the means ± SEM. A 2‐tailed *p* < 0.05 was considered to indicate statistical significance.

## 3 Results

### 3.1 3′tiRNA‐GlyGCC Is Upregulated in Hypoxia

To identify and characterise differentially expressed tsRNAs in hypoxic PH, lung tissues from mice after exposure to hypoxia were selected for Arraystar small RNA microarray analysis. The scatter plot shows the expression and distribution of the tsRNAs in the normoxia (N) and hypoxia (H) groups (Figure 1A). Dysregulated tsRNAs were detected in hypoxic lung tissues (fold change ≥ 1.5 and *p*‐value < 0.05), with 21 upregulated tsRNAs and 20 downregulated tsRNAs (Figure 1B). Moreover, the distributions of different types of dysregulated tsRNAs were analyzed (Figure 1C). We then selected 5 significantly differentially upregulated tsRNAs for further RT–qPCR validation, and the results showed that 3′tiRNA‐GlyGCC was upregulated in hypoxic lung tissues compared with normoxia (Figure 1D). Next, we performed FISH analysis to determine the distribution of 3′tiRNA‐GlyGCC in lung tissues and found that 3′tiRNA‐GlyGCC was mainly expressed in the pulmonary artery smooth muscle layer (Figure 1E). Furthermore, the expression of 3′tiRNA‐GlyGCC was detected in both PASMCs and PAECs, with higher expression in PASMCs exposed to hypoxia (Figure 1F). The subcellular localisation of 3′tiRNA‐GlyGCC was detected via RNA‐FISH, and the results confirmed that 3′tiRNA‐GlyGCC was completely distributed in the cytoplasm of PASMCs (Figure 1G). Therefore, we focused on the role of 3′tiRNA‐GlyGCC in PASMCs, which is important in the development of hypoxic PH, for further research. 3′tiRNA‐GlyGCC, derived from the 3′‐half of mature tRNA‐Gly‐GCC‐6, is a 3′tiRNA with a length of 37 nt (Figure 1H).

**FIGURE 1:**
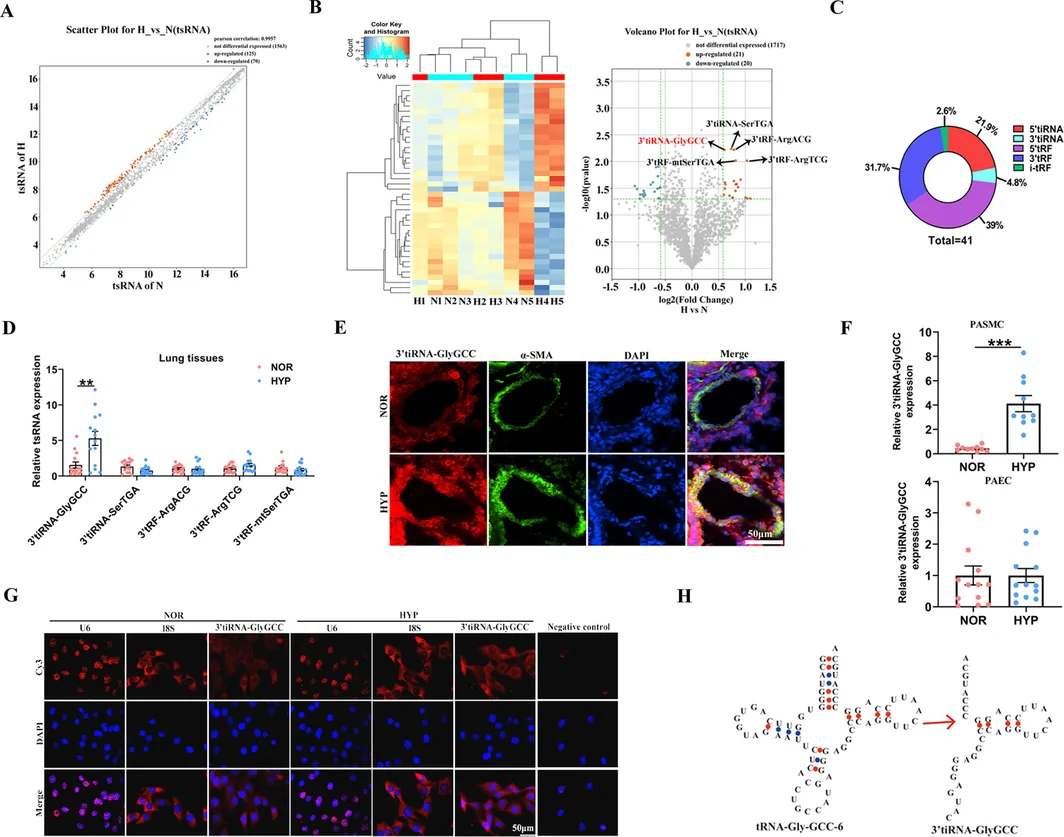
3′Tirna‐GlyGCC is increased in lung and pulmonary artery smooth muscle cell (PASMCs) under hypoxic (HYP) conditions. (A) Scatter plot of tsRNAs in lung tissues from HYP‐exposed mice. (B) Heatmap and volcano plot of tsRNAs in lung tissues from HYP‐exposed mice (fold change ≥ 1.5 and *p*‐value < 0.05). (C) The distribution of tsRNAs subtype numbers. (D) RT‐qPCR analysis of tsRNAs expression in mouse lung (*n* = 14). (E) Fluorescent in situ hybridization (FISH) for 3′tiRNA‐GlyGCC (red) and immunofluorescence (IF) for α‐smooth muscle Actin (α‐SMA, green) in the lung tissues. Scale bars, 50 μm. (F) RT‐qPCR analysis of 3′tiRNA‐GlyGCC in PASMCs and pulmonary artery endothelial cells (PAECs) (*n* = 10–13). (G) Fluorescence in situ hybridization was used to determine the distribution of 3′tiRNA‐GlyGCC in PASMCs. Scale bars, 50 μm. (H) 3′tiRNA‐GlyGCC was derived from mature tRNA‐Gly‐GCC‐6 with a length of 37 nt. All values are presented as the mean ± SEM. Statistical analysis was performed with Student's *t*‐test. DAPI, 2‐(4‐Amidinophenyl)‐6‐indolecarbamidine dihydrochloride; N and NOR, normoxia; H and HYP, hypoxic. **p < 0.01, ***p < 0.001.

### 3.2 Inhibition of 3′tiRNA‐GlyGCC Reverses Hypoxia‐Induced PASMC Proliferation and ERS

To further explore the biological effects of 3′tiRNA‐GlyGCC on the proliferation of PASMCs under hypoxic conditions, we designed and synthesised inhibitors for 3′tiRNA‐GlyGCC. RT‐qPCR results confirmed the inhibitory efficiency (Figure S1A). CCK8 and ethynyl‐2′‐deoxyuridine (EdU) assays were performed, which revealed that the inhibition of 3′tiRNA‐GlyGCC retarded hypoxia‐induced proliferation of PASMCs (Figure 2A,B). Western blot analysis of proliferating cell nuclear antigen (PCNA) and cell cycle‐related proteins (cyclin A, cyclin D) further demonstrated that the inhibition of 3′tiRNA‐GlyGCC significantly attenuated hypoxia‐induced PASMC proliferation (Figure 2C). To investigate the role of 3′tiRNA‐GlyGCC under normoxic conditions, we synthesised a 3′tiRNA‐GlyGCC mimic and confirmed its efficiency by RT‐qPCR (Figure S1B). We then assessed PASMC proliferation and found that 3′tiRNA‐GlyGCC mimics significantly enhanced cell viability, EdU incorporation, and expression of proliferation‐related proteins (Figure S2A–C).

**FIGURE 2:**
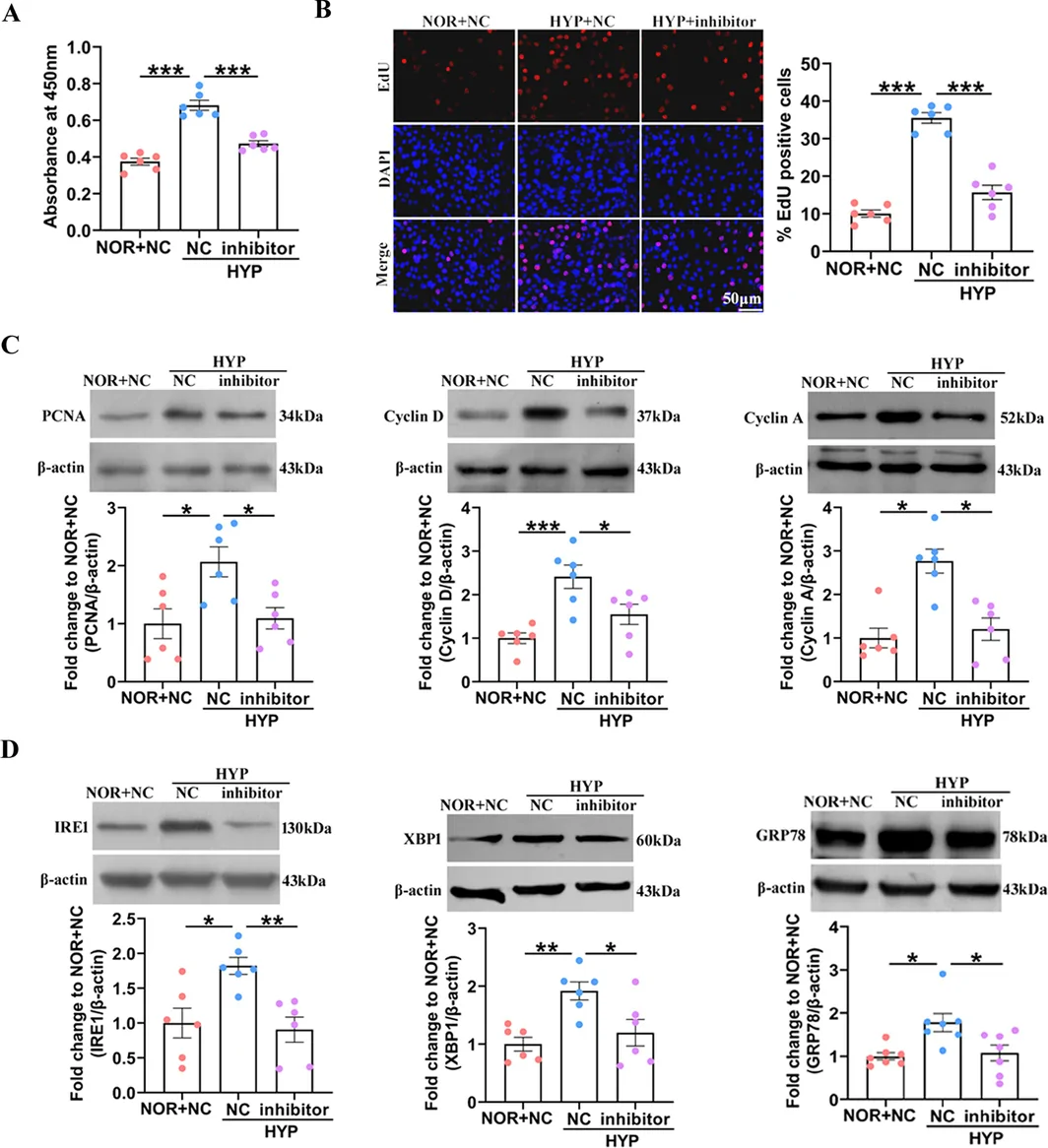
Inhibition of 3′tiRNA‐GlyGCC reversed hypoxia‐induced proliferation and endoplasmic reticulum stress (ERS) of pulmonary arterial smooth muscle cells (PASMCs). (A) CCK8 assay in PASMCs transfected with 3′tiRNA‐GlyGCC inhibitor (*n* = 6). (B) 5‐ethynyl‐2‐deoxyuridine (EdU) assay in PASMCs transfected with 3′tiRNA‐GlyGCC inhibitor (*n* = 6). Scale bars, 50 μm. (C) Representative Western blot and quantification of PCNA, Cyclin D, and Cyclin A in PASMCs transfected with 3′tiRNA‐GlyGCC inhibitor under HYP conditions (*n* = 6). (D) Representative Western blot and quantification of IRE1, XBP1, and GRP78 in PASMCs transfected with 3′tiRNA‐GlyGCC inhibitor under HYP conditions (*n* = 6). All values are presented as the mean ± SEM. Statistical analysis was performed with one‐way ANOVA. NOR, normoxia; HYP, hypoxic; NC, negative control. *p < 0.05, **p < 0.01, ***p < 0.001.

Increasing evidence has demonstrated that ERS occurs during cell proliferation. To evaluate the role of 3′tiRNA‐GlyGCC in ERS in PASMCs, we detected the expression of the marker proteins by western blot after PASMCs were treated with the 3′tiRNA‐GlyGCC inhibitor. The results showed that the inhibition of 3′tiRNA‐GlyGCC significantly reduced the hypoxia‐induced upregulation of ERS marker proteins in the IRE1 pathway, but not in the ATF6 and CHOP pathways (Figure 2D, Figure S3A,B). Additionally, 3′tiRNA‐GlyGCC mimics increased the expression of ERS, including IRE1, XBP1, and GRP78 in PASMCs (Figure S2D,E). Taken together, these findings indicate that upregulation of 3′tiRNA‐GlyGCC under hypoxia promotes excessive proliferation and ERS in PASMCs.

### 3.3 3′tiRNA‐GlyGCC Regulates PASMC Proliferation and ERS via Myrf

To investigate the genes involved in 3′tiRNA‐GlyGCC‐mediated ERS and proliferation, we performed mRNA‐seq in PASMCs. We found that Myrf is a gene regulated by 3′tiRNA‐GlyGCC under hypoxic conditions (Figure 3A). Surprisingly, Myrf is partially located in the endoplasmic reticulum membrane and can maintain endoplasmic reticulum homeostasis [23]. We subsequently verified its expression by RT–qPCR, and the results showed that hypoxia decreased the expression of Myrf, which was reversed by the 3′tiRNA‐GlyGCC inhibitor (Figure 3B). Consistently, the results of Western blot and immunofluorescence assays confirmed that inhibition of 3′tiRNA‐GlyGCC reversed the hypoxia‐induced suppression of Myrf protein expression (Figure 3C,D). To investigate whether the effect of 3′tiRNA‐GlyGCC on proliferation and ERS is dependent on Myrf, we used restorative experiments to confirm these findings. The efficacy of Myrf siRNA transfection is shown in Figure S4A. CCK8 and EdU assays showed that the ability of hypoxia to promote cell proliferation was attenuated by 3′tiRNA‐GlyGCC inhibition, whereas Myrf siRNA reversed this effect (Figure 3E,F). Similar results are also shown in Figure 3G. In addition, Western blot and immunofluorescence experiments showed that treatment with 3′tiRNA‐GlyGCC inhibitor reversed hypoxia‐induced ERS, and the effect was abolished by Myrf knockdown (Figure 3H–J). These results clearly confirmed that 3′tiRNA‐GlyGCC regulates PASMC proliferation and ERS via Myrf under hypoxic conditions.

**FIGURE 3:**
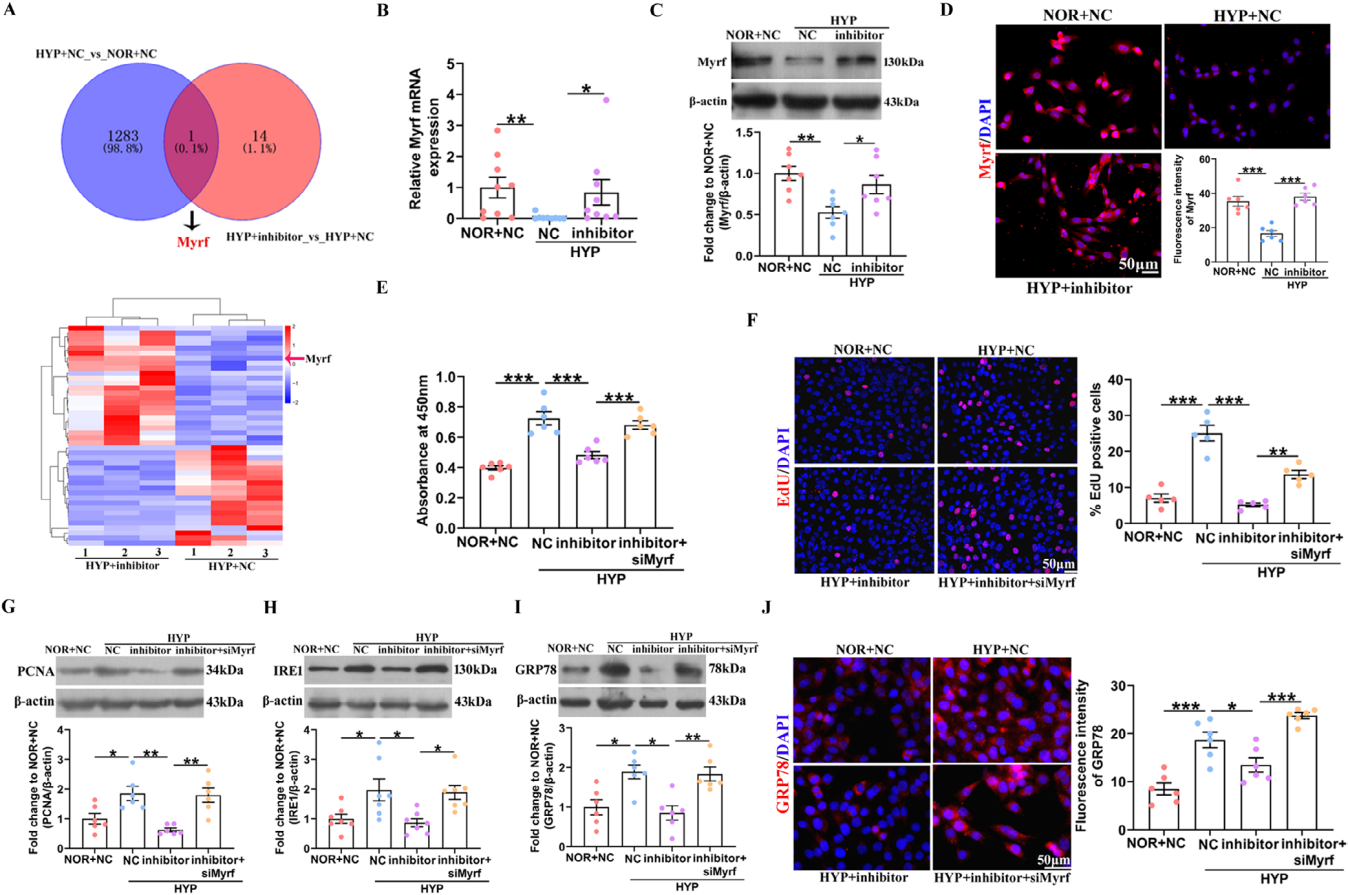
3′tiRNA‐GlyGCC regulated proliferation and endoplasmic reticulum stress (ERS) via myelin regulatory factor (Myrf). (A) RNA‐seq revealed the differentially regulated genes between HYP + 3′tiRNA‐GlyGCC inhibitor group and HYP group (log2FoldChange ≥ 1 and *p*‐value < 0.05). (B) RT‐qPCR analysis showing the effect of inhibition of 3′tiRNA‐GlyGCC on the expressions of the candidate gene Myrf in hypoxic PASMCs (*n* = 9). (C) Representative Western blot and quantification of Myrf in PASMCs transfected with 3′tiRNA‐GlyGCC inhibitor under HYP conditions (*n* = 7). (D) Myrf expression was further examined by immunofluorescence staining (*n* = 6). Scale bars, 50 μm. (E) CCK8 assays in PASMCs transfected with 3′tiRNA‐GlyGCC inhibitor and Myrf siRNA under HYP conditions (*n* = 6). (F) 5‐ethynyl‐2‐deoxyuridine (EdU) assays in PASMCs transfected with 3′tiRNA‐GlyGCC inhibitor and Myrf siRNA under HYP conditions (*n* = 5). Scale bars, 50 μm. (G–I) Representative Western blot and quantification of PCNA, GRP78 and IRE1 in PASMCs transfected with 3′tiRNA‐GlyGCC inhibitor and Myrf siRNA under HYP conditions (*n* = 6). (J) GRP78 expression was examined by immunofluorescence staining (*n* = 6). Scale bars, 50 μm. All values are presented as the mean ± SEM. Statistical analysis was performed with one‐way ANOVA. NOR, normoxia; HYP, hypoxic; NC, negative control. *p < 0.05, **p < 0.01, ***p < 0.001.

### 3.4 3′tiRNA‐GlyGCC Specifically Binds to the 3′untranslated Region (3′UTR) of Myrf mRNA

To investigate the potential molecular mechanism by which 3′tiRNA‐GlyGCC regulates the expression of Myrf, the miRanda website was used to analyse the binding site between the seed region of 3′tiRNA‐GlyGCC and the 3′UTR of Myrf mRNA (Figure 4A). We conducted molecular dynamics (MD) simulation analysis to evaluate the stability of the interaction between 3′tiRNA‐GlyGCC and the Myrf 3′UTR. Root means square fluctuation (RMSF) and radius of gyration (Rg) analyses revealed great stability in the 3′tiRNA‐GlyGCC‐Myrf 3′UTR complex (Figure 4B). RIP analysis showed that 3′tiRNA‐GlyGCC and Myrf mRNA were detectable in the products pulled down by AGO2 (Argonaute 2), a key protein of RNA‐induced silencing complex (Figure 4C). These results suggest that 3′tiRNA‐GlyGCC targets and degrades the Myrf mRNA in the form of a silencing complex. An RNA antisense purification (RAP) assay revealed that Myrf mRNA was significantly enriched in 3′tiRNA‐GlyGCC pull‐down fraction (Figure 4D). Next, a FISH assay showed the colocalization of 3′tiRNA‐GlyGCC with Myrf mRNA in the cytoplasm of PASMCs (Figure 4E). Moreover, a dual‐luciferase assay was used to further verify whether there was an interaction between 3′tiRNA‐GlyGCC and Myrf mRNA. The results confirmed that 3′tiRNA‐GlyGCC suppressed the luciferase activity of the Myrf 3′UTR wild‐type (WT) group but not the Myrf 3′UTR mutant (MUT) group (Figure 4F). These results indicate that 3′tiRNA‐GlyGCC may directly regulate the expression of Myrf mRNA in PASMCs.

**FIGURE 4:**
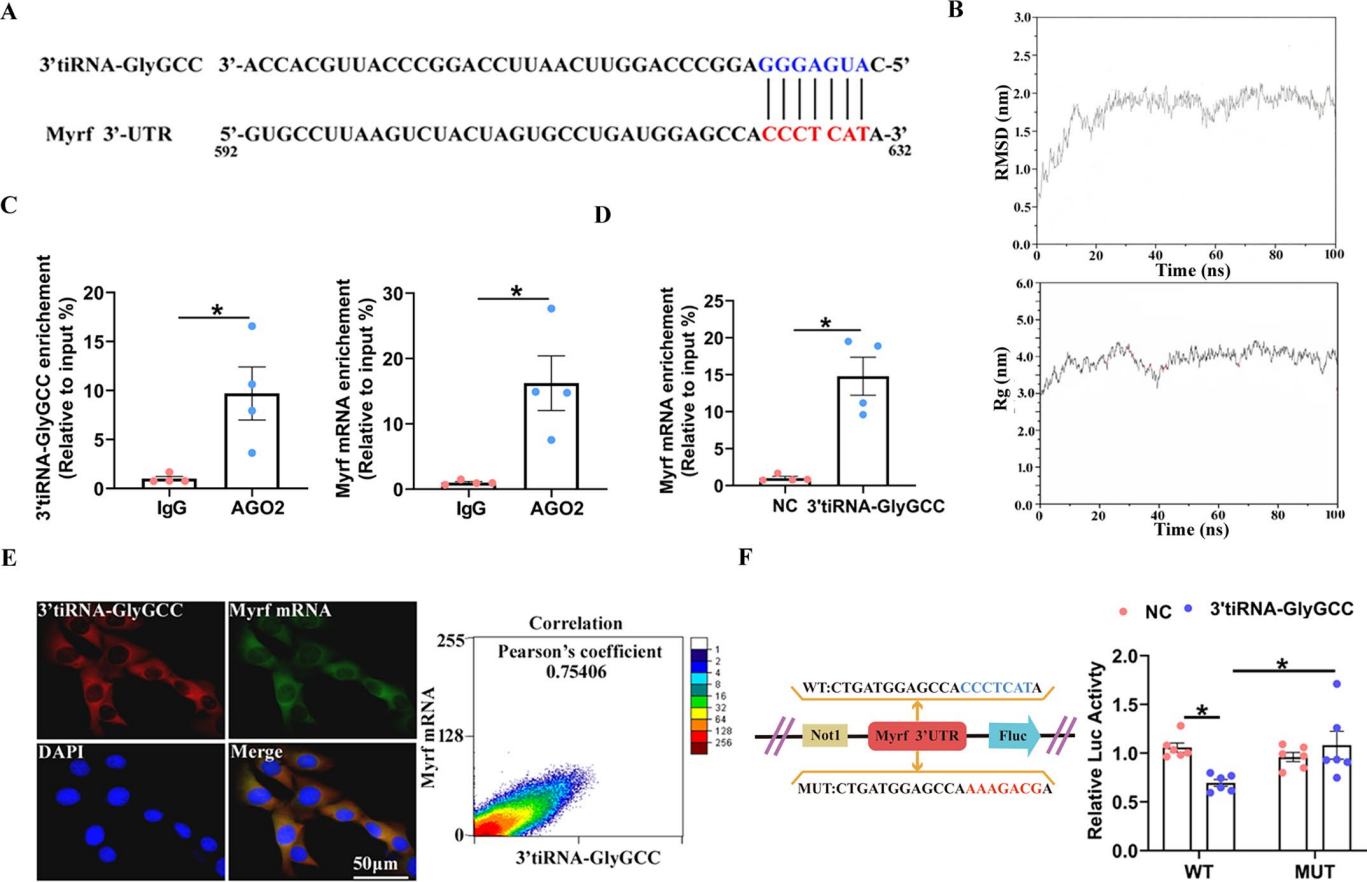
3′tiRNA‐GlyGCC specifically targeted and bound to the 3'untranslated region (UTR) of Myrf mRNA. (A) MiRanda software predicts binding site between the seed region of 3′tiRNA‐GlyGCC and the 3'UTR of Myrf mRNA. (B) Root mean square fluctuation (RMSF) and radius of gyration (Rg) analysis showed stability of 3′tiRNA‐GlyGCC‐Myrf 3'UTR complex. (C) RIP assays showing that the immunoprecipitation product of AGO2 detected the expression of 3′tiRNA‐GlyGCC and Myrf mRNA in PASMCs (*n* = 4). (D) RAP assays showing that the biotin‐labelled 3′tiRNA‐GlyGCC detected the expression of Myrf mRNA in PASMCs (*n* = 4). (E) Cell FISH assays showing that 3′tiRNA‐GlyGCC colocalized with Myrf mRNA in PASMCs. Scale bars, 50 μm. (F) Dual‐luciferase assays were used to validate the interactions between 3′tiRNA‐GlyGCC and Myrf mRNA in 293 T cells (*n* = 6). All values are presented as the mean ± SEM. Statistical analysis was performed with Student's *t*‐test. NOR, normoxia; HYP, hypoxic; NC, negative control. *p < 0.05.

### 3.5 3′tiRNA‐GlyGCC Interacts With Eef1a1 to Regulate Stability of Myrf mRNA

Recent studies have shown that tsRNAs can also be involved in disease progression by binding to target proteins. To further explore the underlying mechanism by which 3′tiRNA‐GlyGCC regulates ERS and proliferation in PASMC, RNA‐pulldown and mass spectrometry (MS) assays were performed to identify 3′tiRNA‐GlyGCC‐associated proteins. We selected Eef1a1, which was highly ranked and upregulated in hypoxic PASMCs [24], for further study (Figure 5A,B). To verify this interaction, RNA‐pulldown and RIP experiments were performed, and the results confirmed that 3′tiRNA‐GlyGCC directly binds to Eef1a1 (Figure 5C,D). Consistent with these findings, an immunofluorescence assay showed that 3′tiRNA‐GlyGCC colocalized with Eef1a1 in PASMCs (Figure 5E). We then predicted and visualised the 3‐dimensional structural docking of 3′tiRNA‐GlyGCC–Eef1a1, and the results demonstrated that 3′tiRNA has multiple binding sites on Eef1a1 (Figure 5F). To verify which domain of Eef1a1 interacts with 3′tiRNA‐GlyGCC, we constructed Flag‐tagged Eef1a1 domain truncations and conducted RNA‐pulldown assays. The results demonstrated that 3′tiRNA‐GlyGCC binds to the GTP‐binding domain and the Tu C‐terminal domain of Eef1a1 (Figure 5G). Furthermore, as shown in Figure 5H,I, the expression of Eef1a1 increased under hypoxia; however, inhibition of 3′tiRNA‐GlyGCC did not affect Eef1a1 expression.

**FIGURE 5:**
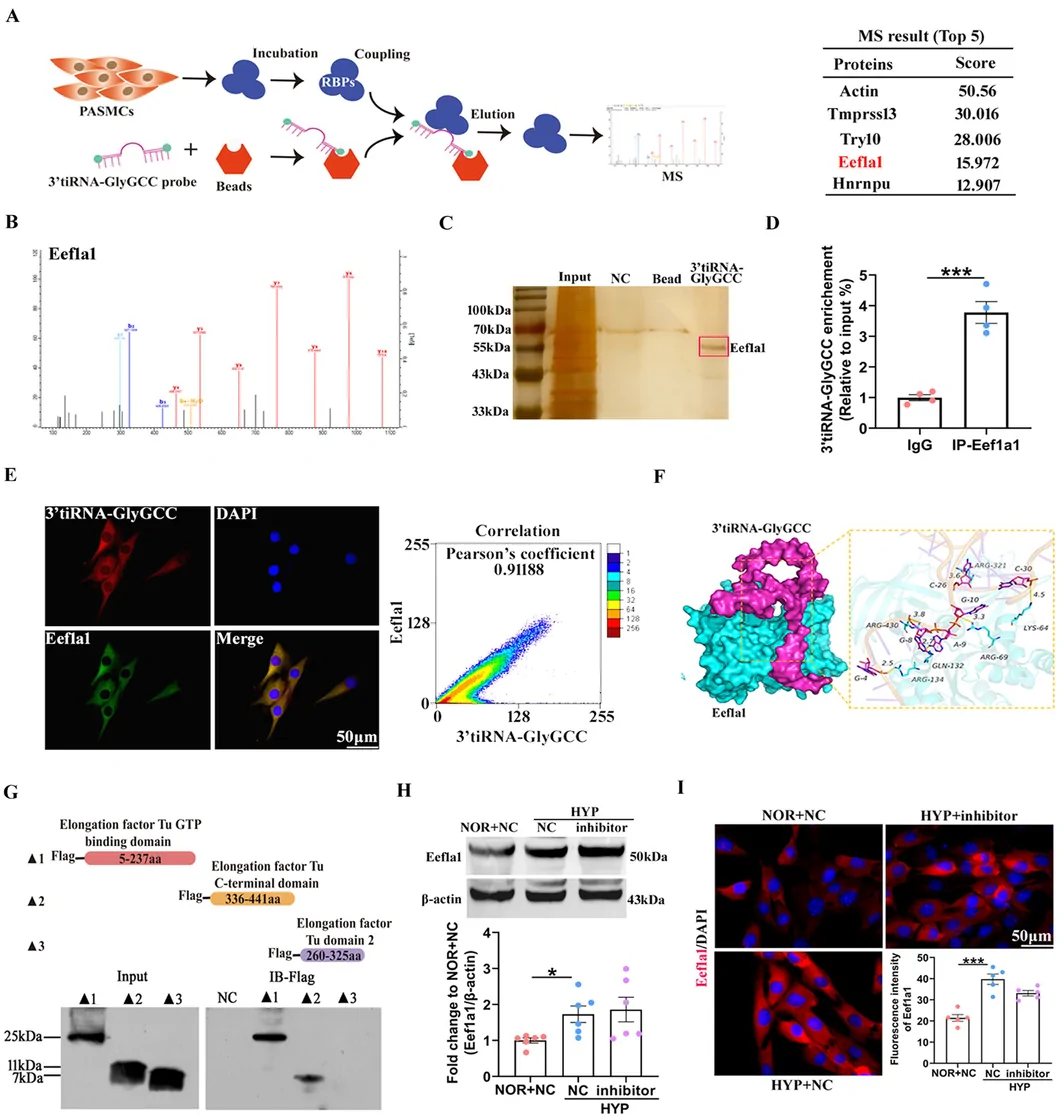
3′tiRNA‐GlyGCC interacted with eukaryotic translation elongation factor 1 alpha 1 (Eef1a1). (A) The workflow and MS result (Top 5) of 3′tiRNA‐GlyGCC pulldown assay. (B) The mass spectrum of the secondary structure of Eef1a1. (C) Silver staining assay was performed to detect the differential proteins obtained by the pulldown assay in PASMCs. (D) RIP assay was performed to detect the interaction of 3′tiRNA‐GlyGCC and Eef1a1 in PASMCs (*n* = 4). (E) Cell FISH assays showing that 3′tiRNA‐GlyGCC colocalized with Eef1a1 in PASMCs. Scale bars, 50 μm. (F) Schrodinger2019.01 software predicted and visualised the 3‐dimensional structural docking of 3′tiRNA‐GlyGCC (magenta) and Eef1a1 (cyan). (G) RNA pulldown verified the interaction between 3′tiRNA‐GlyGCC and structural domain of Eef1a1 in 293 T cells. (H) Representative Western blot and quantification of Eef1a1 in PASMCs transfected with 3′tiRNA‐GlyGCC inhibitor under HYP conditions (*n* = 6). (I) Eef1a1 expression was further examined by immunofluorescence staining (*n* = 5). Scale bars, 50 μm. All values are presented as the mean ± SEM. Statistical analysis was performed with one‐way ANOVA or Student's *t*‐test. NOR, normoxia; HYP, hypoxic; NC, negative control. *p < 0.05, ***p < 0.001.

Next, to clarify the detailed mechanism by which the 3′tiRNA‐GlyGCC‐Eef1a1 axis functions, we investigated the effects of this axis on Myrf. Eef1a1 is known to exert its functions by regulating mRNA stability. Therefore, we predicted the binding probability of Eef1a1 with the 3′UTR and 5′UTR of Myrf mRNA, and the results revealed that Eef1a1 exhibits stronger binding to the 3′UTR region of Myrf mRNA (Figure 6A). catRAPID analysis predicted that Eef1a1 could interact with the 3′UTR of Myrf mRNA (Figure 6B). Furthermore, RIP assays demonstrated that the level of the Eef1a1‐Myrf mRNA 3'UTR complex was higher in the 3′tiRNA‐GlyGCC‐inhibited group than in the NC group under hypoxia (Figure 6C). Consistent with these results, FISH and immunofluorescence staining colocalization assays showed that colocalization of Eef1a1 and Myrf mRNA was increased in the 3′tiRNA‐GlyGCC‐inhibited group compared with the NC group under hypoxia (Figure 6D). We then detected the effect of the 3′tiRNA‐GlyGCC‐Eef1a1 axis on the expression of Myrf. RT–qPCR and immunofluorescence analysis revealed that inhibition of 3′tiRNA‐GlyGCC reversed the hypoxia‐induced downregulation of Myrf mRNA and protein, which was reduced by Eef1a1 silencing (Figure S4B, Figure 6E,F). Subsequently, a Myrf mRNA stability assay was performed. The results showed that hypoxia significantly accelerated the degradation of Myrf mRNA, whereas inhibition of 3′tiRNA‐GlyGCC markedly prolonged Myrf mRNA stability in hypoxic PASMCs, and Eef1a1 silencing abrogated this stabilising effect (Figure 6G). Together, these data demonstrate that 3′tiRNA‐GlyGCC regulates Myrf mRNA stability by recruiting Eef1a1 to the 3′UTR of Myrf mRNA.

**FIGURE 6:**
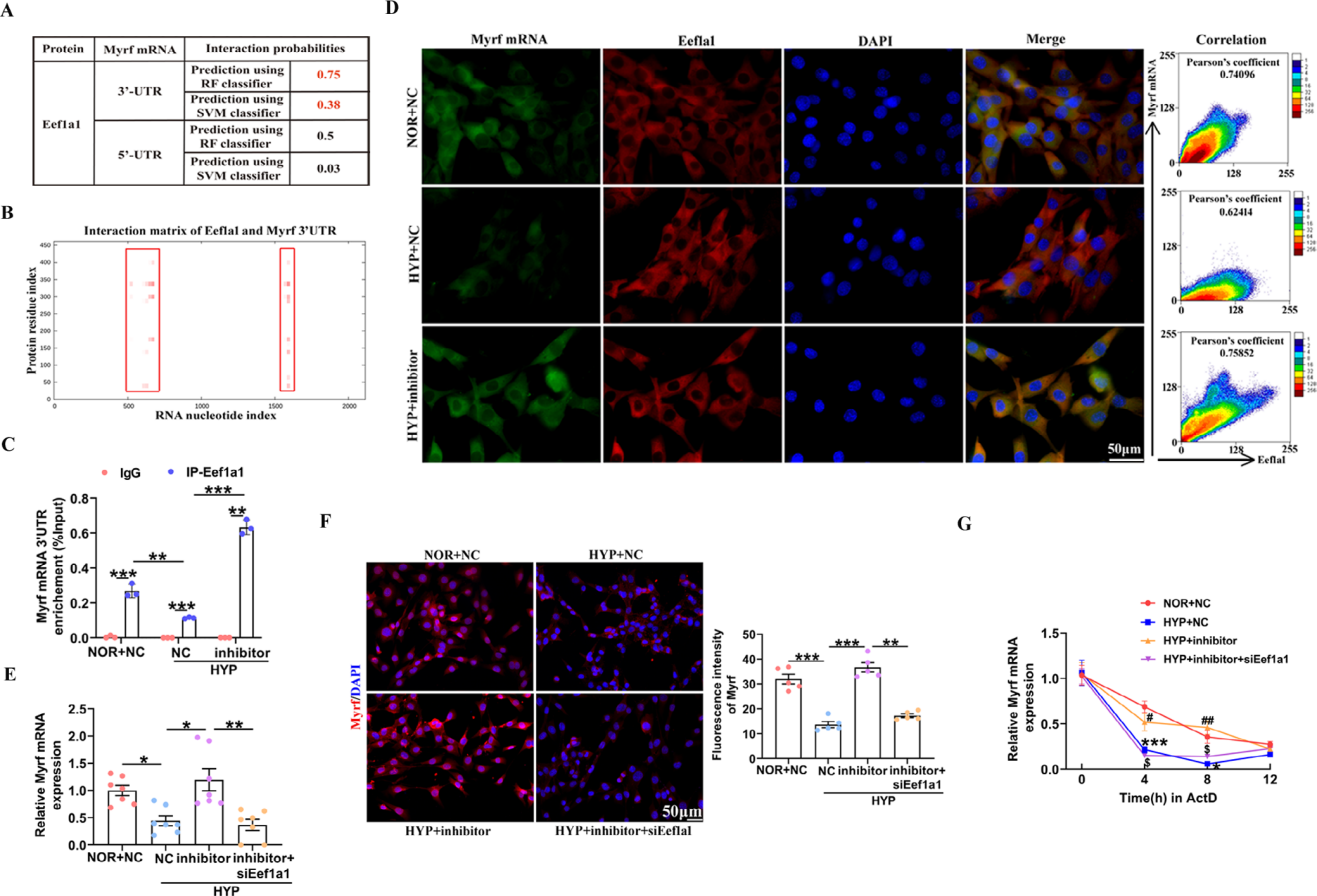
3′tiRNA‐GlyGCC affected the binding of Eef1a1 to 3'untranslated region (UTR) of Myrf mRNA. (A) RPISeq website (http://pridb.gdcb.iastate.edu/RPISeq/index.html) predicted the interaction probabilities of Eef1a1 to the 3'UTR and 5'UTR of Myrf mRNA. (B) catRAPID website (http://s.tartaglialab.com/page/catrapid_group) predicted the binding sites of Eef1a1 to the 3'UTR of Myrf mRNA. (C) RIP assay was performed to detect the interaction of Eef1a1 and 3'UTR of Myrf mRNA in PASMCs (*n* = 3). (D) Immunofluorescence was used to observe the colocalization of Eef1a1 and 3'UTR of Myrf mRNA in the cytoplasm. Scale bar, 50 μm. (E) Myrf mRNA level was examined by RT‐qPCR assays in PASMCs transfected with 3′tiRNA‐GlyGCC inhibitor and Eef1a1 siRNA under HYP conditions (*n* = 7). (F) Myrf expression was further examined by immunofluorescence staining (*n* = 5). Scale bars, 50 μm. (G) The RT‐qPCR analysis of Myrf mRNA levels after ActD (10 μm) treatment at 0 h, 4 h, 8 h and 12 h (*n* = 8). All values are presented as the mean ± SEM. Statistical analysis was performed with one‐way ANOVA or two‐way ANOVA. NOR, normoxia; HYP, hypoxic; NC, negative control. *^,$,#^ *p* < 0.05, **^,##^ *p* < 0.01, ***p < 0.001.

### 3.6 Angiogenin (Ang) Participates in Hypoxic PASMC ERS and Proliferation by Regulating the Expression of 3′tiRNA‐GlyGCC

The ribonuclease angiogenin (Ang) has been associated with the production of tiRNAs [25]. To explore the production of 3′tiRNA‐GlyGCC, we first detected the expression of Ang in hypoxic PASMCs. The results showed that Ang expression was increased in PASMCs under hypoxic conditions (Figure 7A). We transfected Ang siRNA into PASMCs to silence Ang, and the interference efficiency is shown in Figure S4C. Subsequently, we examined the expression of 3′tiRNA‐GlyGCC, and the results suggested that 3′tiRNA‐GlyGCC was significantly downregulated when Ang was knocked down (Figure 7B). Next, we performed rescue experiments to determine whether Ang is involved in the ERS and proliferation by regulating 3′tiRNA‐GlyGCC. CCK8 and EdU assays showed that silencing of Ang partially suppressed the hypoxia‐induced proliferation of PASMCs, whereas the 3′tiRNA‐GlyGCC mimic reversed this effect (Figure 7C,D). Similar results were observed in the Western blot experiment (Figure 7E). In addition, PASMC transfection with siAng decreased the protein expression of GRP78 under hypoxia, an effect that was reversed by the 3′tiRNA‐GlyGCC mimic (Figure 7F,G). These results suggest that Ang, an upstream regulator of 3′tiRNA‐GlyGCC, contributes to PASMC ERS and proliferation by regulating 3′tiRNA‐GlyGCC expression.

**FIGURE 7:**
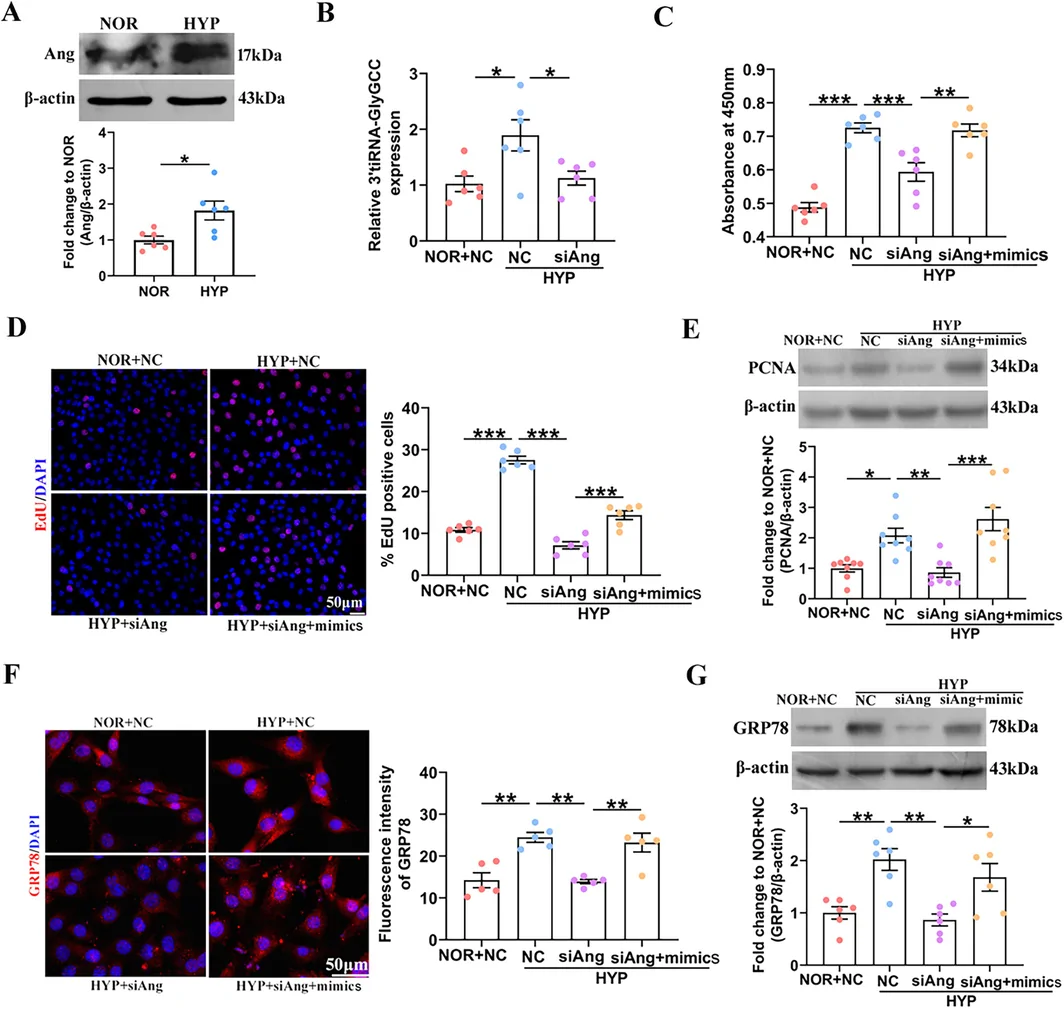
Angiogenin (Ang) participates in hypoxic PASMC ERS and proliferation by regulating the expression of 3′tiRNA‐GlyGCC. (A) Representative Western blot and quantification of Ang in hypoxic PASMCs (*n* = 6). (B) 3′tiRNA‐GlyGCC level was examined by RT‐qPCR assays in PASMCs transfected with Ang siRNA under HYP conditions (*n* = 6). (C) CCK8 assays in PASMCs transfected with Ang siRNA and 3′tiRNA‐GlyGCC mimic under HYP conditions (*n* = 6). (D) 5‐ethynyl‐2‐deoxyuridine (EdU) assays in PASMCs transfected with Ang siRNA and 3′tiRNA‐GlyGCC mimic under HYP conditions (*n* = 6). Scale bars, 50 μm. (E) Representative Western blot and quantification of PCNA in PASMCs transfected with Ang siRNA and 3′tiRNA‐GlyGCC mimic under HYP conditions (*n* = 8). (F) GRP78 expression was examined by immunofluorescence staining (*n* = 5). Scale bars, 50 μm. (G) Representative Western blot and quantification of GRP78 in PASMCs transfected with Ang siRNA and 3′tiRNA‐GlyGCC mimic under HYP conditions (*n* = 6). All values are presented as the mean ± SEM. Statistical analysis was performed with one‐way ANOVA or Student's *t*‐test. NOR, normoxia; HYP, hypoxic; NC, negative control. *p < 0.05, **p < 0.01, ***p < 0.001.

### 3.7 Inhibition of 3′tiRNA‐GlyGCC Alleviates the Pathological Process of SuHx‐Induced PH In Vivo

To further investigate the functional significance of 3′tiRNA‐GlyGCC in vivo, we constructed an AAV9 (adeno‐associated virus vector 9) to inhibit 3′tiRNA‐GlyGCC in a SuHx‐induced mouse model of PH (Figure 8A). Quantitative reverse transcription PCR analysis confirmed that 3′tiRNA‐GlyGCC was effectively knocked down in lung tissues infected with AAV9 (Figure S5). Inhibition of 3′tiRNA‐GlyGCC markedly reduced right ventricular systolic pressure (RVSP) and right ventricular hypertrophy induced by SuHx (Figure 8B,C). Additionally, the 3′tiRNA‐GlyGCC inhibitor significantly improved the impaired pulmonary artery acceleration time (PAAT) and pulmonary artery velocity‐time integral (PAVTI) in the SuHx mice, without affecting left heart function (LVEF) (Figure 8D). Haematoxylin and eosin (HE) staining and Western blot for PCNA showed that the inhibition of 3′tiRNA‐GlyGCC alleviated SuHx‐induced pulmonary vascular remodelling and proliferation (Figure 8E,F). Immunofluorescence staining of GRP78 revealed that the inhibition of 3′tiRNA‐GlyGCC reduced SuHx‐induced ERS (Figure 8G). Moreover, the 3′tiRNA‐GlyGCC inhibitor restored Myrf expression, which was confirmed by immunofluorescence assay (Figure 8H). The above results suggest that 3′tiRNA‐GlyGCC plays an important role in the pathological process of SuHx‐induced PH.

**FIGURE 8:**
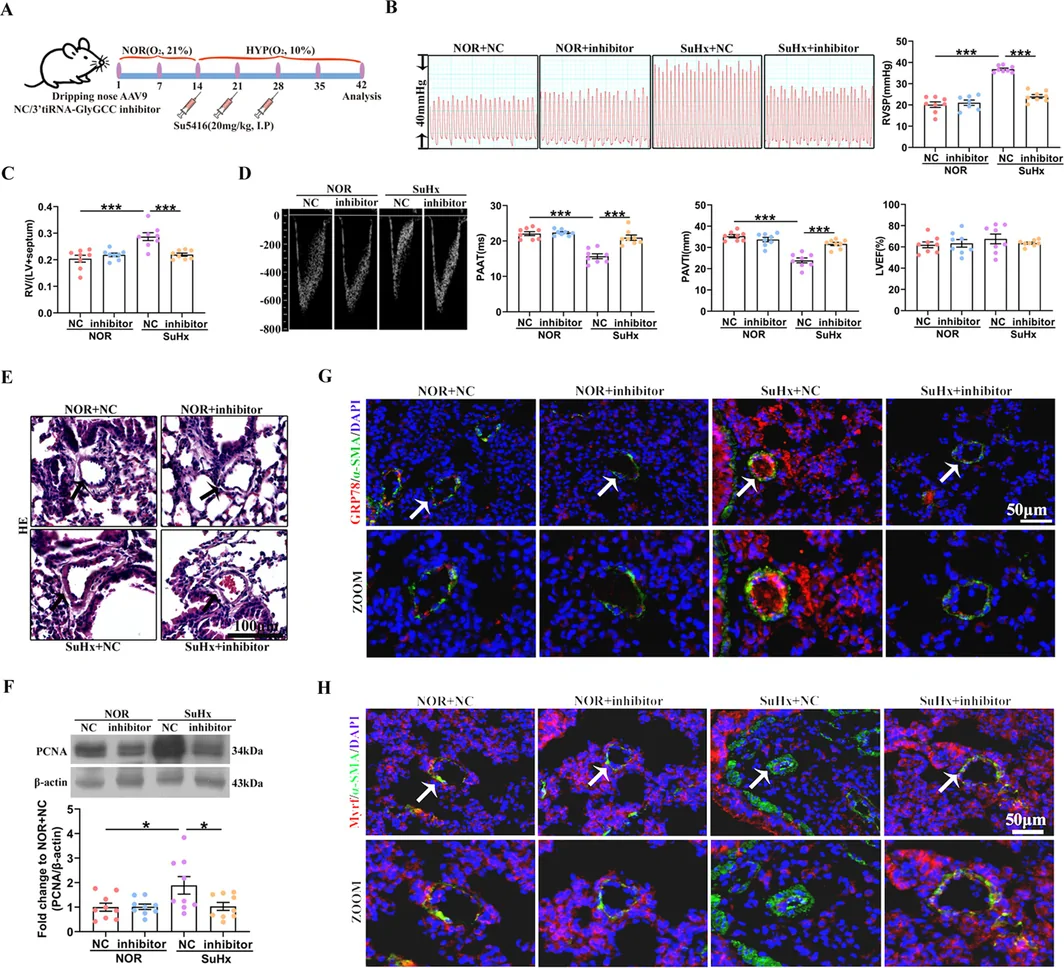
Inhibition of 3′tiRNA‐GlyGCC reversed the pathological process of pulmonary hypertension (PH) in vivo. (A) Schematic illustration showing the drug treatment protocol. (B, C) Right ventricular systolic pressure and right ventricular/left ventricular + septum weight ratio in the SuHx‐induced PH mice models (*n* = 8). (D) Pulmonary artery velocity time integral, pulmonary artery acceleration time and left ventricular ejection fraction of the SuHx‐induced PH mice models infected with AAV9‐NC and AAV9‐3′tiRNA‐GlyGCC (*n* = 8). (E) Pulmonary arterial morphological analysis was performed by using haematoxylin and eosin (HE) staining. Scale bars, 100 μm. (F) Representative Western blot and quantification of PCNA in lung tissues (*n* = 8). (G, H) Immunofluorescence of GRP78, Myrf and α‐smooth muscle Actin (α‐SMA) in mouse lung sections. Scale bars, 50 μm. All values are presented as the mean ± SEM. Statistical analysis was performed with one‐way ANOVA. NOR, normoxia; HYP, hypoxic; NC, negative control. *p < 0.05, ***p < 0.001.

## 4 Discussion

Currently, the functional role of tiRNAs in PH remains largely unknown. In the present study, for the first time, we identified 3′tiRNA‐GlyGCC as a novel regulator in PASMCs and found that 3′tiRNA‐GlyGCC is highly expressed in hypoxic PASMCs and contributes to the progression of PH. As shown in Figure 9, we demonstrated that Ang promotes the expression of 3′tiRNA‐GlyGCC, thereby inducing ERS and the proliferation of PASMCs. In terms of mechanism, 3′tiRNA‐GlyGCC targets Myrf mRNA and reduces its expression. Furthermore, the binding of 3′tiRNA‐GlyGCC to Eef1a1 is enhanced, which reduces the binding of Eef1a1 to the 3'UTR of Myrf mRNA and consequently suppresses Myrf expression. Our data reveal the functional roles and regulatory mechanism of 3′tiRNA‐GlyGCC in PH pathogenesis, which could serve as a potential target for the treatment of PH.

**FIGURE 9:**
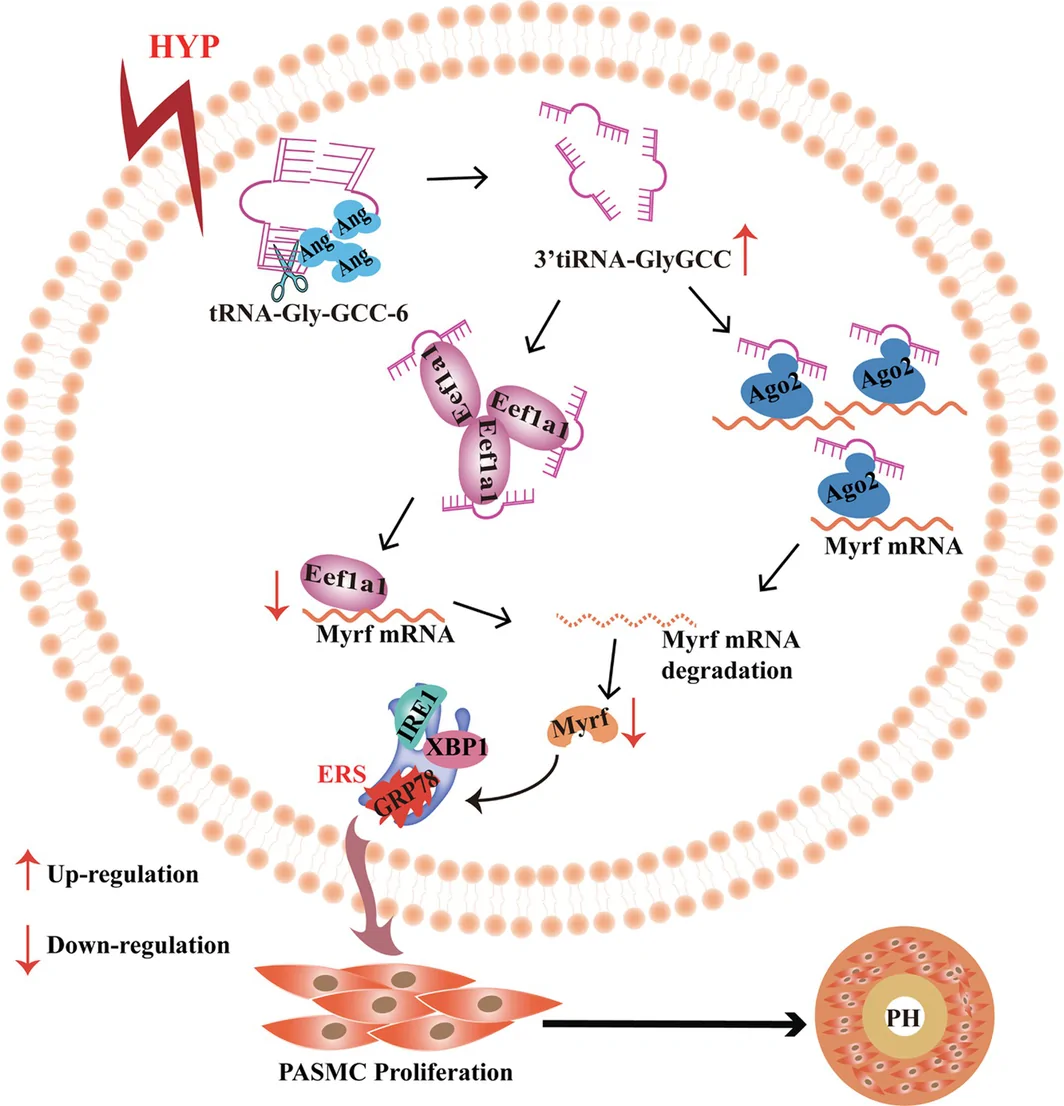
Proposed mechanism for the role of 3′tiRNA‐GlyGCC in pulmonary hypertension (PH). Ang directly cleaved tRNA‐Gly‐GCC‐6 and promoted the production of 3'tiRNA‐GlyGCC in hypoxic PASMCs. On one hand, 3'tiRNA‐GlyGCC interacted with Eef1a1 to interfere with the binding of Eef1a1 to the 3′‐untranslated region (UTR) of Myrf mRNA, thereby reducing Myrf mRNA stability. On the other hand, 3'tiRNA‐GlyGCC directly targeted and bound to the 3'UTR of Myrf mRNA to repress Myrf expression. Ultimately, downregulation of Myrf resulted in enhanced ERS, increased PASMC proliferation, and the progression of PH.

tsRNAs, which were initially considered random degradation products of tRNAs, are now widely recognised as small noncoding RNAs with important biological functions [26, 27]. Notably, tsRNAs have been implicated in the regulation of neurological disorders, cardiovascular diseases, and tumours [28, 29, 30]. Emerging evidence indicates that tRF‐1‐AspGTC regulates hypoxia‐induced PASMC proliferation and apoptosis resistance, revealing that tsRNA‐tRF plays crucial roles in the development of hypoxia‐induced PH [20]. However, the role of tiRNAs in the occurrence and development of PH remains unexplored. In this study, to explore the role of tiRNAs in PH, we conducted Arraystar small RNA microarray analysis and found that 3′tiRNA‐GlyGCC was significantly upregulated. Notably, we further demonstrated that hypoxia‐induced upregulation of 3′tiRNA‐GlyGCC was specific to PASMCs, whereas no significant alteration was observed in PAECs. This cell‐specific expression pattern suggests that 3′tiRNA‐GlyGCC may act as a PASMC‐predominant regulator rather than a ubiquitous stress‐responsive molecule in the pulmonary vasculature. Functionally, inhibition of 3′tiRNA‐GlyGCC effectively alleviated hypoxia‐induced PH in mice. However, to eliminate the inherent biological differences between mice and humans, further studies are needed to determine the expression of 3′tiRNA‐GlyGCC in clinical samples from patients with PH.

ERS is a crucial determinant of PASMC proliferation and pulmonary vascular remodelling. Our previous study also highlighted the crucial roles of circSSR1 and BCAT1 in hypoxia‐induced PASMC ERS [31, 32]. However, our knowledge of the regulatory factors related to ERS is only at the tip of the iceberg, and further research is still needed. In the current study, we found that the inhibition of 3′tiRNA‐GlyGCC markedly reduced the expression of IRE1, GRP78, and XBP1, without affecting the levels of ATF6 or CHOP, indicating that 3′tiRNA‐GlyGCC selectively modulates the IRE1 signalling branch of ERS rather than the ATF6 or PERK/CHOP pathways. These findings identify 3′tiRNA‐GlyGCC as a novel inducer of ERS in hypoxic PASMCs. To the best of our knowledge, this is the first study to demonstrate that a tiRNA mediates ERS through selective regulation of the IRE1 pathway in PASMCs, revealing a new regulatory layer in hypoxic pulmonary vascular remodelling.

According to our results, 3′tiRNA‐GlyGCC regulated IRE1‐mediated ERS through two parallel regulatory pathways: on the one hand, 3′tiRNA‐GlyGCC directly targets the Myrf 3′UTR to inhibit its translation; on the other hand, 3′tiRNA‐GlyGCC indirectly regulates Myrf mRNA stability by targeting Eef1a1. Both pathways are simultaneously activated upon the induction of 3′tiRNA‐GlyGCC under hypoxia, and coordinately downregulate Myrf expression. In terms of temporal dominance during hypoxia progression, we speculate that the direct targeting pathway may act more rapidly and dominate in the early stage of hypoxia, when 3′tiRNA‐GlyGCC is quickly elevated. In contrast, the Eef1a1‐mediated indirect pathway may gradually become prominent at later hypoxic stages, when the expression levels of 3′tiRNA‐GlyGCC and Eef1a1 reach a sustained steady state. Such stage‐specific dominance may allow cells to fine‐tune Myrf expression dynamically, matching the progressive changes in ERS and cellular adaptation during hypoxic pulmonary vascular remodelling.

Studies have demonstrated that Myrf is cleaved proteolytically into an N‐terminal region with transcriptional activity and a C‐terminal region located in the endoplasmic reticulum [33]. In addition, Myrf can alleviate ERS and maintain endoplasmic reticulum homeostasis in cancer cells [23], suggesting a possible relationship between Myrf and PH. To our surprise, in this study, we found that 3′tiRNA‐GlyGCC could significantly regulate the expression levels of Myrf in hypoxic PASMCs. Thus, we hypothesised that Myrf affects ERS in PASMC under hypoxic conditions. However, it remains unclear how Myrf regulates IRE1‐mediated ERS—whether through its transcriptionally active N‐terminal domain, or via its C‐terminal domain located in the endoplasmic reticulum. We speculate that Myrf may regulate the IRE1 signalling pathway through multiple potential ways: first, Myrf may directly bind to the promoter regions of IRE1 to promote its transcription, thereby activating the IRE1‐XBP1 cascade; in addition, Myrf may interact with IRE1 to regulate its phosphorylation, ubiquitination, or subcellular localisation, further modulating the activation status of the signalling pathway. Considering that IRE1 signalling is closely related to PASMC proliferation and vascular remodelling, clarifying the specific molecular mechanism by which Myrf regulates the IRE1 pathway will be a meaningful direction for future research.

Eef1a1 plays an important role in peptide elongation during protein translation in eukaryotic cells [34]. Increasing evidence suggests that Eef1a1 is also involved in many diseases through multiple mechanisms, including binding DNA as a transcription factor and binding mRNA as a stabiliser [35, 36]. Importantly, Eef1a1 also promotes the ferroptosis of PASMCs via the upregulation of ACSL4 caused by hypoxia [24]. In the present study, we identified Eef1a1 as a novel interacting protein of 3′tiRNA‐GlyGCC, providing a new layer of regulatory mechanism underlying ERS and hypoxic PH. Notably, inhibition of 3′tiRNA‐GlyGCC did not alter the total protein level of Eef1a1, implying that 3′tiRNA‐GlyGCC may modulate Eef1a1 function through protein–RNA interaction rather than affecting its expression. Further RIP assays, immunofluorescence colocalization, and RT‐qPCR confirmed that Eef1a1 binds to the 3′UTR of Myrf mRNA and regulates its stability in hypoxic PASMCs. These findings suggest that the 3′tiRNA‐GlyGCC–Eef1a1–Myrf axis may participate in hypoxia‐induced functional remodelling of PASMCs, including ferroptosis and ERS, thereby contributing to the pathogenesis of hypoxic PH.

## 5 Conclusions

In conclusion, we demonstrated for the first time that 3′tiRNA‐GlyGCC is a critical regulator of PH development and progression, providing new insights into the mechanisms underlying PH pathogenesis.

## Author Contributions

L.Z., X.W. and C.M. designed the research and wrote the paper; L.Z., X.G., X.Z., Y.H., Y.X, H.Y, D.G., H.D. and J.L. carried out the study and performed the experiments; W.C. and X.W. conceived, designed, and supervised the study. All the authors read and examined the manuscript.

## Funding

This work was supported by the National Natural Science Foundation of China [82400062, 32400949, 82570079], Heilongjiang Provincial Natural Science Foundation of China [LH2024H029], the Fundamental Research Funds for the Provincial Universities [JFQN202301], Guidance project of Daqing city [zdy‐2025‐108], the High‐quality Development Science and Technology Program of Xiamen Municipal Health Commission [2024GZL‐CX05], and the Science and Technology Project of the Health Commission of Fujian Province [2025CXAO63].

## Ethics Statement

All animal experiments complied with the ethical standards of the 1964 Declaration of Helsinki and its later amendments and were approved by the Ethics Committees of Harbin Medical University (HMUDQ20250723001).

## Consent

The authors have nothing to report.

## Conflicts of Interest

The authors declare no conflicts of interest.

## Supporting information


**Figure S1:** Efficiency of inhibitors and mimics of 3′tiRNA‐GlyGCC (*n* = 5). All values are presented as the mean ± SEM. Statistical analysis was performed with Student's *t*‐test. NC, negative control. *p < 0.05, **p < 0.01.
**Figure S2:** 3′tiRNA‐GlyGCC mimics increased proliferation and endoplasmic reticulum stress (ERS) of pulmonary arterial smooth muscle cells (PASMCs). (A) CCK8 assays in PASMCs transfected with 3′tiRNA‐GlyGCC mimics (*n* = 6). (B) 5‐ethynyl‐2‐deoxyuridine (EdU) assays in PASMCs transfected with 3′tiRNA‐GlyGCC mimics (*n* = 6). Scale bars, 50 μm. (C) Representative Western blot and quantification of PCNA, Cyclin D and Cyclin A in PASMCs transfected with 3′tiRNA‐GlyGCC mimics (*n* = 6). (D) Representative Western blot and quantification of IRE1, and XBP1 in PASMCs transfected with 3′tiRNA‐GlyGCC mimics (*n* = 6). (E) GRP78 expression was examined by immunofluorescence staining (*n* = 6). Scale bars, 50 μm. All values are presented as the mean ± SEM. Statistical analysis was performed with Student's *t*‐test. NC, negative control. *p < 0.05, ***p < 0.001.
**Figure S3:** (A, B) Inhibition of 3′tiRNA‐GlyGCC did not reduce the increase in ATF6 and CHOP ERS pathway marker proteins caused by hypoxia (*n* = 6). All values are presented as the mean ± SEM. Statistical analysis was performed with one‐way ANOVA. NOR, normoxia; HYP, hypoxic; NC, negative control. *p < 0.05, **p < 0.01.
**Figure S4:** Interference efficiency of Myrf, Eef1a1 and Ang (*n* = 6). All values are presented as the mean ± SEM. Statistical analysis was performed with one‐way ANOVA. NC, negative control; si, siRNA. *p < 0.05, **p < 0.01.
**Figure S5:** Interference efficiency of 3′tiRNA‐GlyGCC in lung tissues (*n* = 8). All values are presented as the mean ± SEM. Statistical analysis was performed with one‐way ANOVA. NC, negative control; si, siRNA. *p < 0.05, **p < 0.01, ***p < 0.001.
**Table S1:** RT‐qPCR primer sequence.
**Table S2:** siRNA sequences.
**Table S3:** Probe sequences.

**Data S1:** cpr70238‐sup‐0002‐Supinfo02.docx.

## Data Availability

The datasets used and/or analyzed during the current study are available from the corresponding author on reasonable request.
