# Supplementary material for: A Specific tRNA Half, 3'tiRNA‐GlyGCC, Regulates Hypoxic Pulmonary Artery Smooth Muscle Cell Proliferation via Myrf‐Mediated Endoplasmic Reticulum Stress
Source: Cell Prolif. 2026 May 29;59(11):e70238. doi: 10.1111/cpr.70238 (PMC13326036; doi:10.1111/cpr.70238)
Supplement: Supplementary file 1 [file CPR-59-e70238-s001.docx]

**Uncropped blots**

Figure 2C

Figure 2D

Figure 3

Figure 5H

Figure 7A

Figure 7E, G

Figure 8F

Figure S2

Figure S3

Figure S4
