# Supplementary material for: A Specific tRNA Half, 3'tiRNA‐GlyGCC, Regulates Hypoxic Pulmonary Artery Smooth Muscle Cell Proliferation via Myrf‐Mediated Endoplasmic Reticulum Stress
Source: Cell Prolif. 2026 May 29:e70238. Online ahead of print. doi: 10.1111/cpr.70238 (PMC13326036; doi:10.1111/cpr.70238)
Supplement: Supplementary file 1 — Figure S1: Efficiency of inhibitors and mimics of 3′tiRNA‐GlyGCC (n = 5). All values are presented as the mean ± SEM. Statistical analysis was performed with Student's t‐test. NC, negative control. *p < 0.05, **p < 0.01. Figure S2: 3′tiRNA‐GlyGCC mimics increased proliferation and endoplasmic reticulum stress (ERS) of pulmonary arterial smooth muscle cells (PASMCs). (A) CCK8 assays in PASMCs transfected with 3′tiRNA‐GlyGCC mimics (n = 6). (B) 5‐ethynyl‐2‐deoxyuridine (EdU) assays in PASMCs transfected with 3′tiRNA‐GlyGCC mimics (n = 6). Scale bars, 50 μm. (C) Representative Western blot and quantification of PCNA, Cyclin D and Cyclin A in PASMCs transfected with 3′tiRNA‐GlyGCC mimics (n = 6). (D) Representative Western blot and quantification of IRE1, and XBP1 in PASMCs transfected with 3′tiRNA‐GlyGCC mimics (n = 6). (E) GRP78 expression was examined by immunofluorescence staining (n = 6). Scale bars, 50 μm. All values are presented as the mean ± SEM. Statistical analysis was performed with Student's t‐test. NC, negative control. *p < 0.05, ***p < 0.001. Figure S3: (A, B) Inhibition of 3′tiRNA‐GlyGCC did not reduce the increase in ATF6 and CHOP ERS pathway marker proteins caused by hypoxia (n = 6). All values are presented as the mean ± SEM. Statistical analysis was performed with one‐way ANOVA. NOR, normoxia; HYP, hypoxic; NC, negative control. *p < 0.05, **p < 0.01. Figure S4: Interference efficiency of Myrf, Eef1a1 and Ang (n = 6). All values are presented as the mean ± SEM. Statistical analysis was performed with one‐way ANOVA. NC, negative control; si, siRNA. *p < 0.05, **p < 0.01. Figure S5: Interference efficiency of 3′tiRNA‐GlyGCC in lung tissues (n = 8). All values are presented as the mean ± SEM. Statistical analysis was performed with one‐way ANOVA. NC, negative control; si, siRNA. *p < 0.05, **p < 0.01, ***p < 0.001. Table S1: RT‐qPCR primer sequence. Table S2: siRNA sequences. Table S3: Probe sequences. [file CPR-9999-e70238-s001.docx]

Figure S1


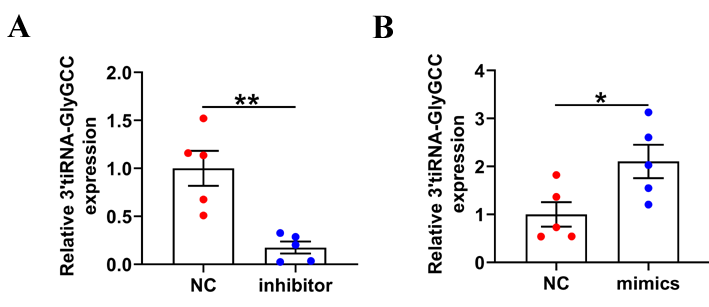


Figure S1. Efficiency of inhibitors and mimics of 3´tiRNA-GlyGCC (n=5). All values are presented as the mean±SEM. Statistical analysis was performed with Student's t-test. NC, negative control. **P*<0.05, ***P*<0.01.

Figure S2


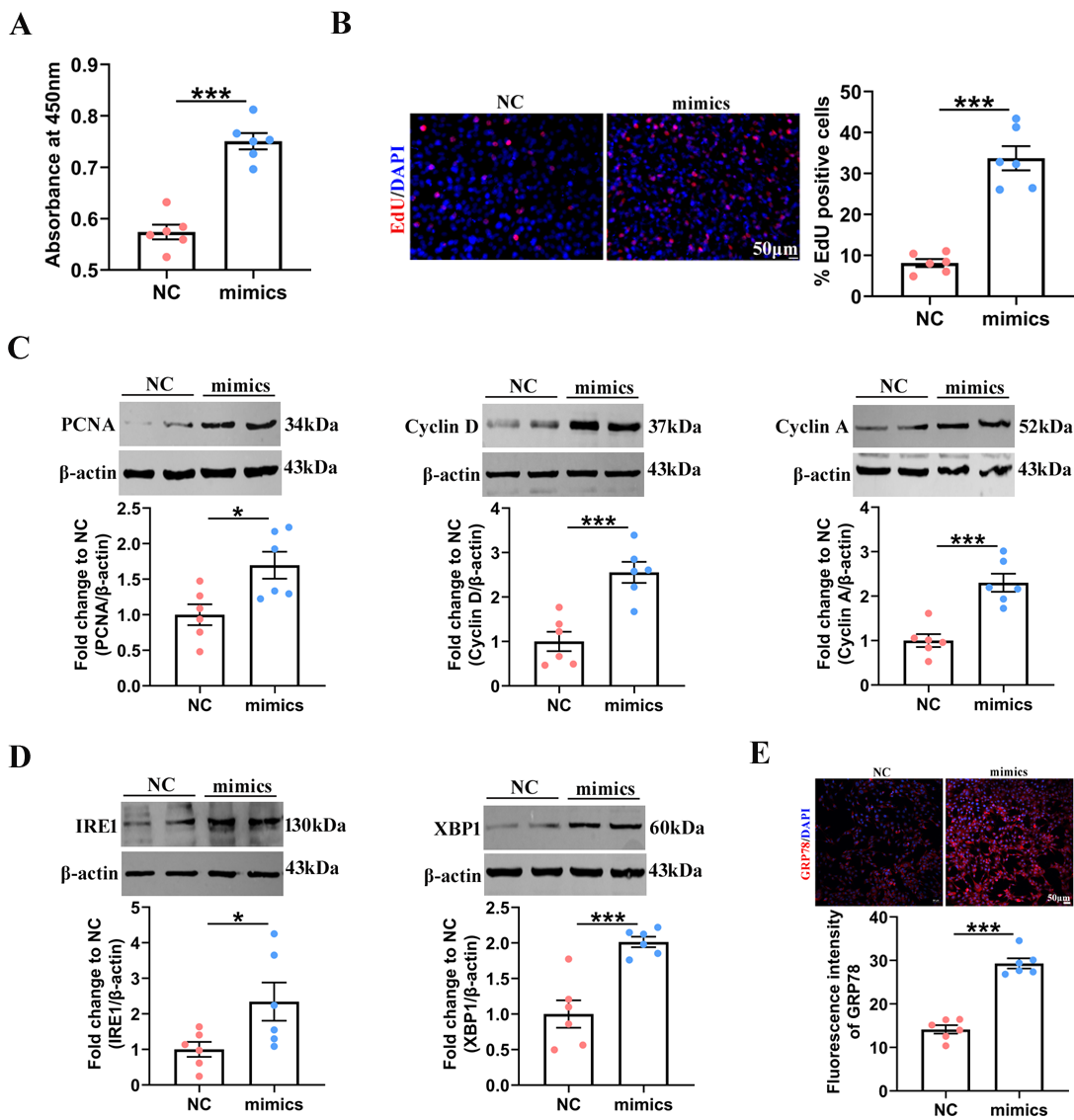


Figure S2. 3´tiRNA-GlyGCC mimics increased proliferation and endoplasmic reticulum stress (ERS) of pulmonary arterial smooth muscle cells (PASMCs). **A** CCK8 assays in PASMCs transfected with 3´tiRNA-GlyGCC mimics (n=6). **B** 5-ethynyl-2-deoxyuridine (EdU) assays in PASMCs transfected with 3´tiRNA-GlyGCC mimics (n=6). Scale bars, 50 µm. **C** Representative Western blot and quantification of PCNA, Cyclin D and Cyclin A in PASMCs transfected with 3´tiRNA-GlyGCC mimics (n=6). **D** Representative Western blot and quantification of IRE1, and XBP1 in PASMCs transfected with 3´tiRNA-GlyGCC mimics (n=6). **E** GRP78 expression was examined by immunofluorescence staining (n=6). Scale bars, 50 µm. All values are presented as the mean±SEM. Statistical analysis was performed with Student's t-test. NC, negative control. **P*<0.05, ****P*<0.001.

Figure S3


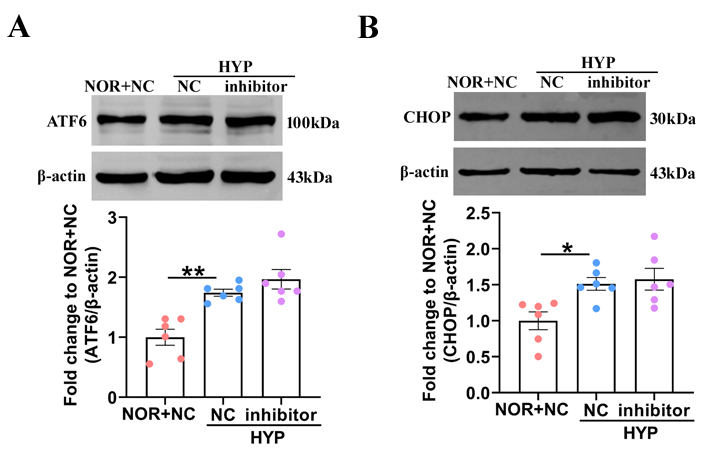


Figure S3. **A, B** Inhibition of 3´tiRNA-GlyGCC did not reduce the increase in ATF6 and CHOP ERS pathway marker proteins caused by hypoxia (n=6). All values are presented as the mean±SEM. Statistical analysis was performed with one-way ANOVA. NOR, normoxia; HYP, hypoxic; NC, negative control. **P*<0.05, ***P*<0.01.

Figure S4


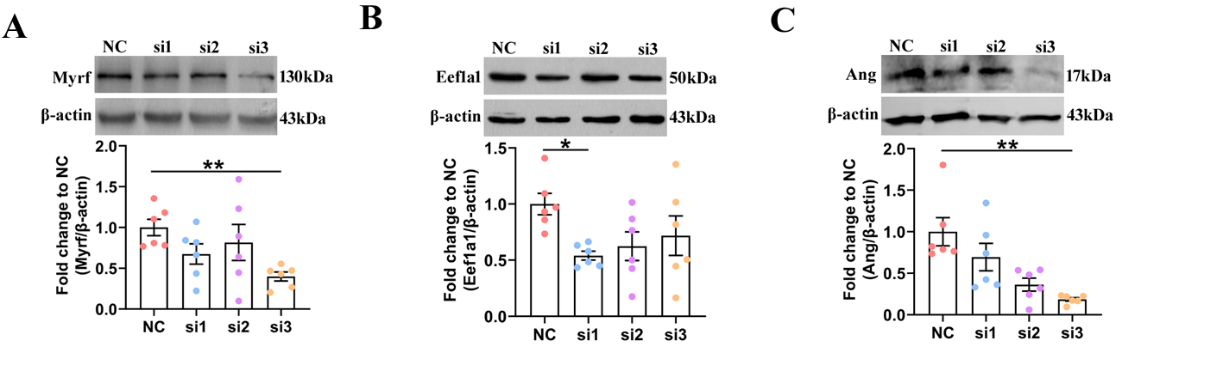


Figure S4. Interference efficiency of Myrf, Eef1a1 and Ang (n=6). All values are presented as the mean±SEM. Statistical analysis was performed with one-way ANOVA. NC, negative control; si, siRNA. **P*<0.05, ***P*<0.01.

Figure S5


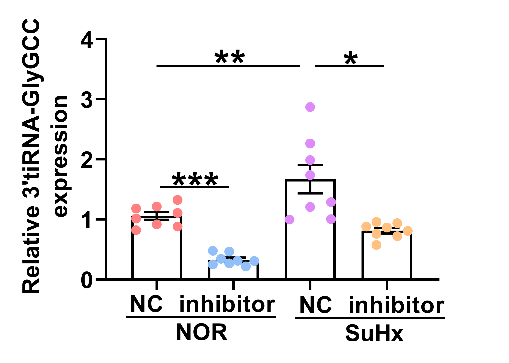


Figure S5. Interference efficiency of 3´tiRNA-GlyGCC in lung tissues (n=8). All values are presented as the mean±SEM. Statistical analysis was performed with one-way ANOVA. NC, negative control; si, siRNA. **P*<0.05, ***P*<0.01, ****P*<0.001.

Table S1: RT-qPCR primer sequence

| **Gene Name** | **Forward primer** | **Reverse primer** |
| --- | --- | --- |
| 3’tiRNA-GlyGCC | GAGGCCCAGGTTCAATTCCA | TATGGTTGTTCACGACTCCTTCAC |
| 3’tRF-ArgTCG | GCGTCATCGAATCCCTTCGT | TATGGTTGTTCACGACTCCTTCAC |
| 3’tiRNA-SerTGA | GCGCAGGTTCGAACCCTG | TATGGTTGTTCACGACTCCTTCAC |
| 3’tRF-ArgACG | CTGCTGTTCTCCTACCTGGCTC | TATGGTTGTTCACGACTCCTTCAC |
| 3’tRF-mtSerTGA | CTGCTGTTTCGATTCCTTCCTT | TATGGTTGTTCACGACTCCTTCAC |
| Myrf | GCCCAGATCTCAGAGCGTAT | TGGTGGAAGACCGTATCTGG |

Table S2: siRNA sequences

| **Gene Name** | **Sense（5'-3'）** | **Antisense（5'-3'）** |
| --- | --- | --- |
| Myrf-1 | GCAACAGCAUGGAGCUGAATT | UUCAGCUCCAUGCUGUUGCTT |
| Myrf-2 | GCUGCAUGGUGUGAAGCUATT | UAGCUUCACACCAUGCAGCTT |
| Myrf-3 | GGUGUCUAUGUCCACACUATT | UAGUGUGGACAUAGACACCTT |
| Eef1a1-1 | GCUGCUGGUGUUGGUGAAUTT | AUUCACCAACACCAGCAGCTT |
| Eef1a1-2 | GCUGGAGCCAAGUGCUAAUTT | AUUAGCACUUGGCUCCAGCTT |
| Eef1a1-3 | CCACAUAGCAUGCAAGUUUTT | AAACUUGCAUGCUAUGUGGTT |
| Ang-1 | GCAACAAGAGCAACAUCAATT | UUGAUGUUGCUCUUGUUGCTT |
| Ang-2 | GGACGACAGAUACUGUGAATT | UUCACAGUAUCUGUCGUCCTT |
| Ang-3 | GGGUUCAGACAUGUUGUUATT | UAACAACAUGUCUGAACCCTT |
| 3’tiRNA-GlyGCC inhibitor | UGGUGCAAUGGGCCUGGAAUUGAACCUGGGCCUCCCUCAUG | |
| 3’tiRNA-GlyGCC mimic | CATGAGGGAGGCCCAGGTTCAATTCCAGGCCCATTGCACCA | |

Table S3: Probe sequences

| **Gene Name** | **Sequences（5'-3'）** |
| --- | --- |
| 3’tiRNA-GlyGCC | TGGT+GCAATGG+GCCTGGAATTGAA+CCTGGGCC+TCCCTCATG |
| Myrf 3’UTR | AGCA+ACTCCAG+TGTAAGGAC+TTCCAG+TCTC |
