# Supplementary material for: A Specific tRNA Half, 3'tiRNA‐GlyGCC, Regulates Hypoxic Pulmonary Artery Smooth Muscle Cell Proliferation via Myrf‐Mediated Endoplasmic Reticulum Stress
Source: Cell Prolif. 2026 May 29:e70238. Online ahead of print. doi: 10.1111/cpr.70238 (PMC13326036; doi:10.1111/cpr.70238)

**Uncropped blots**

Figure 2C


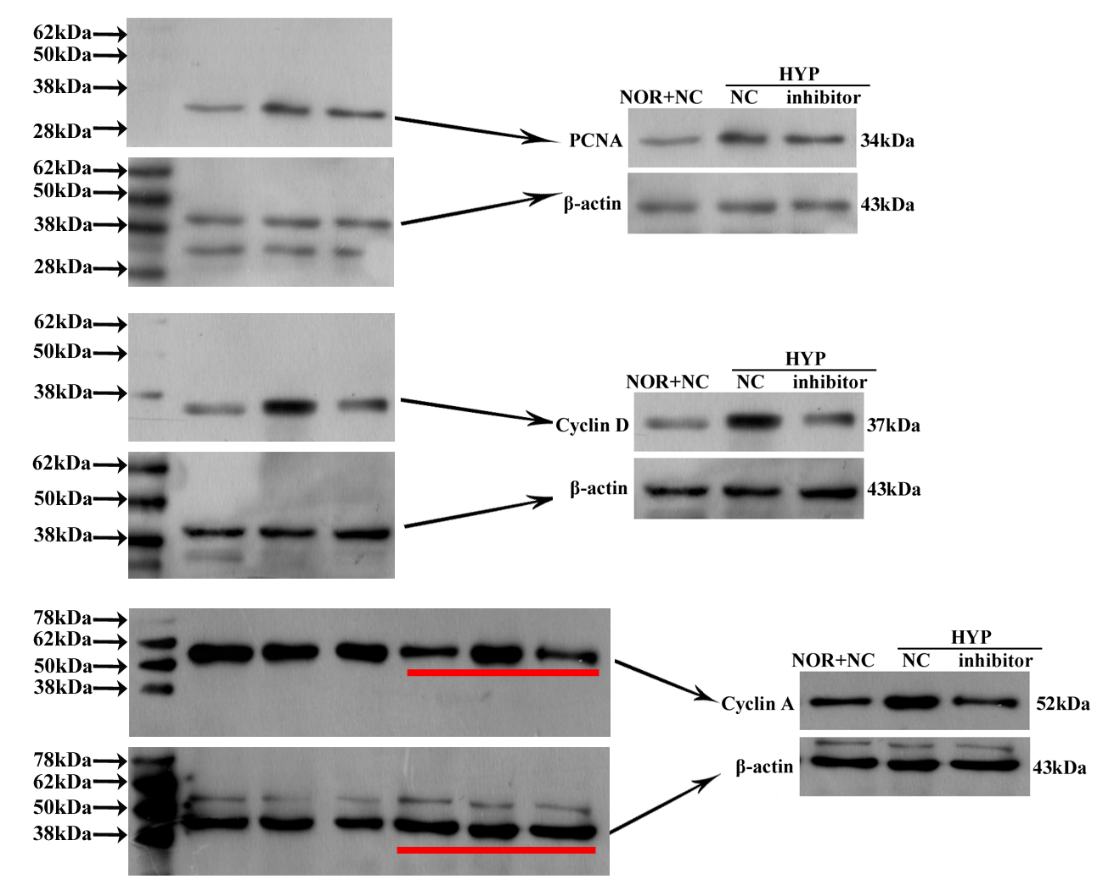


Figure 2D


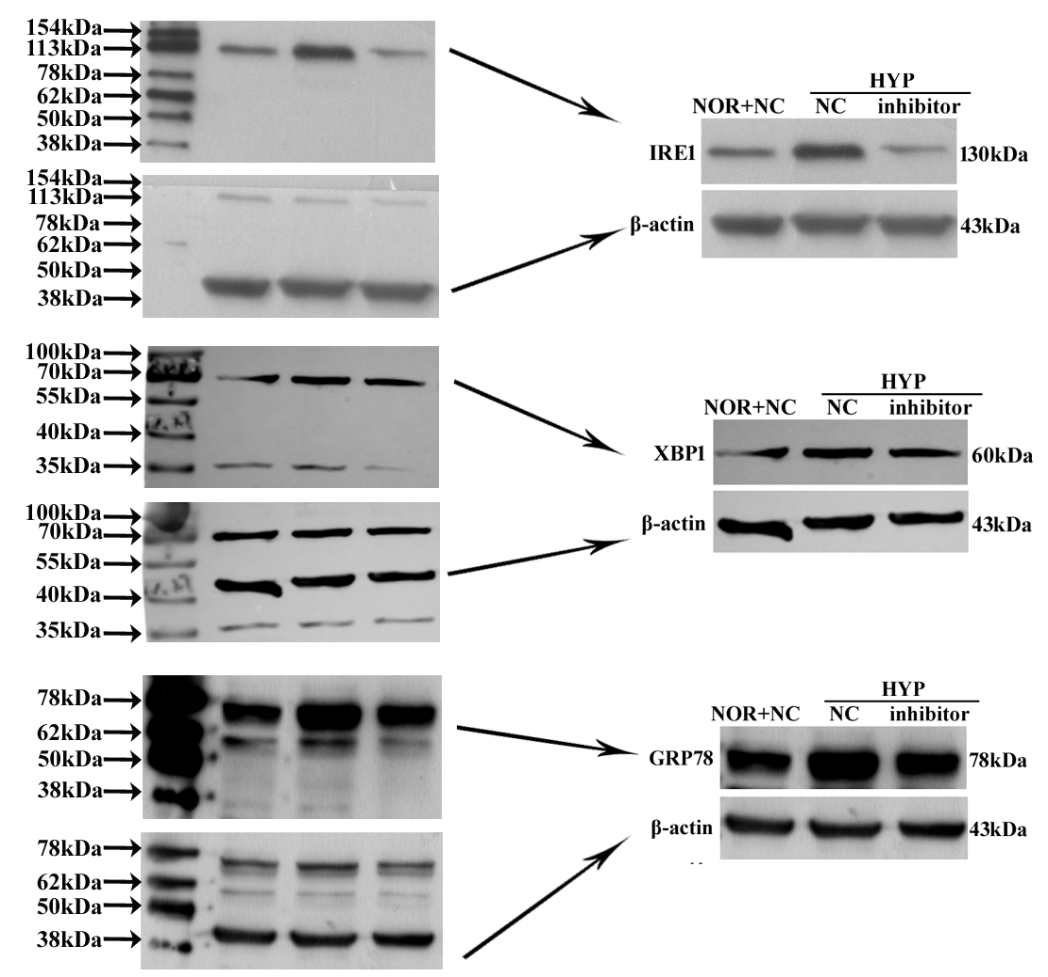


Figure 3


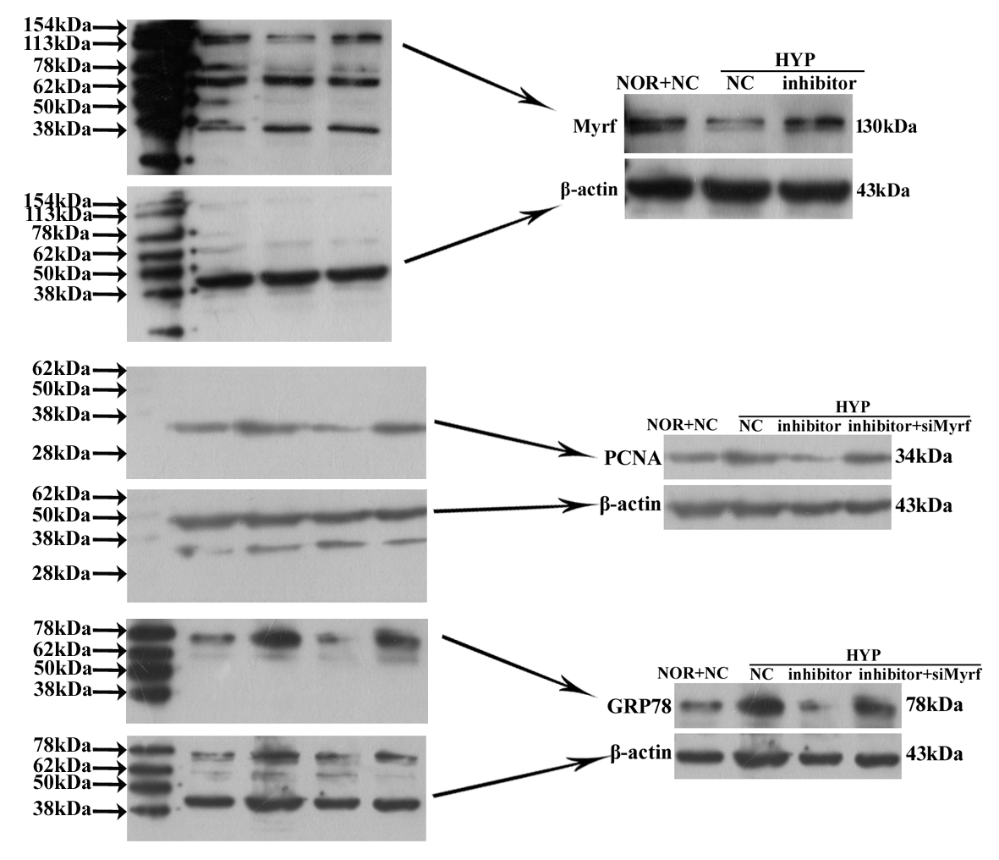


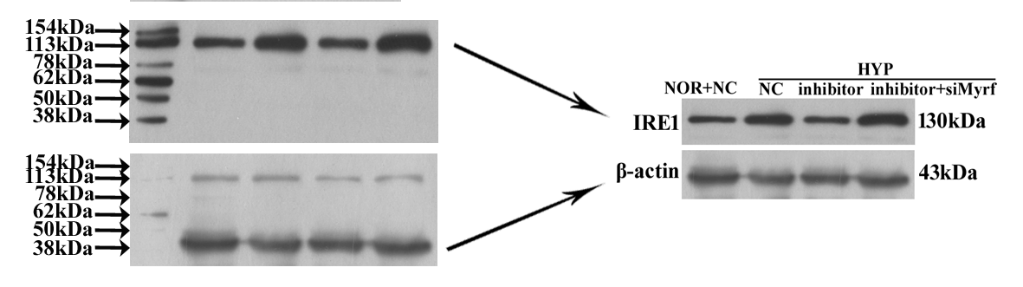


Figure 5H


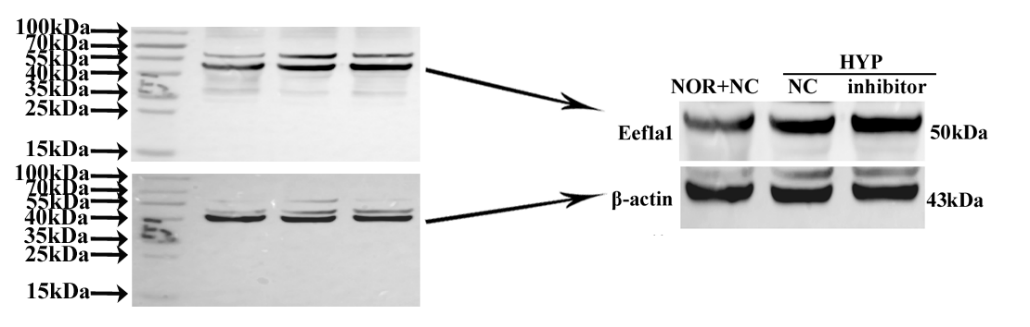


Figure 7A


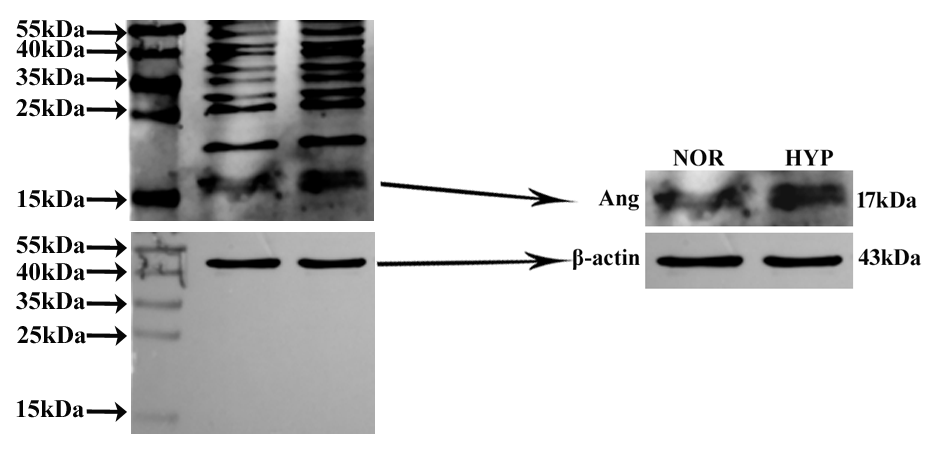


Figure 7E, G


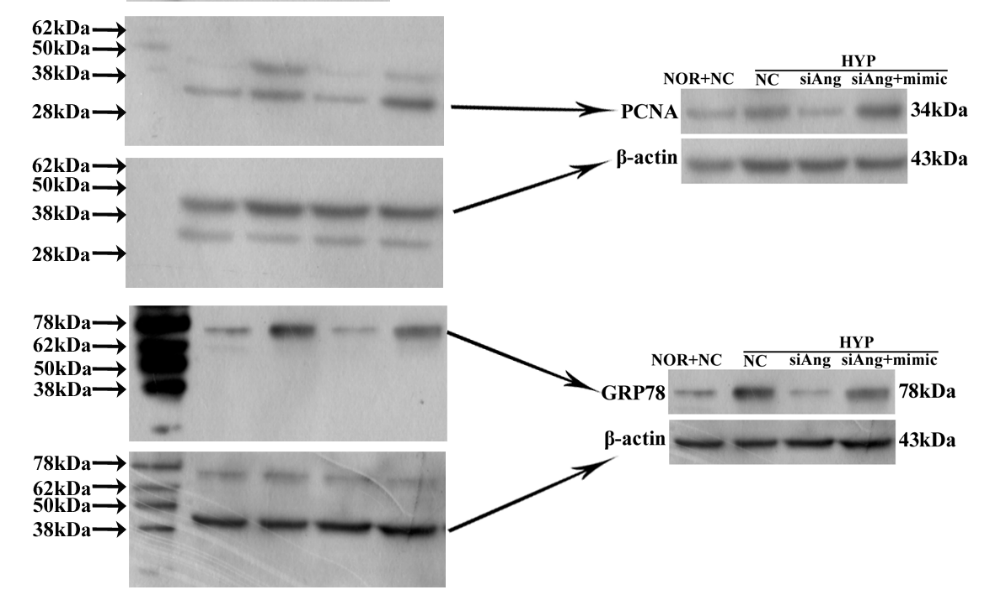


Figure 8F


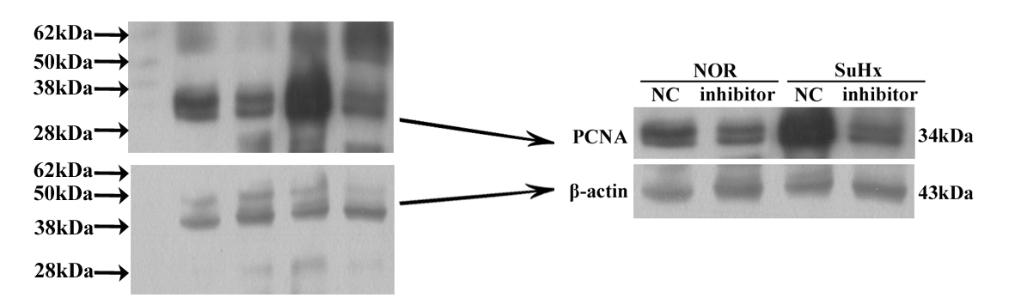


Figure S2


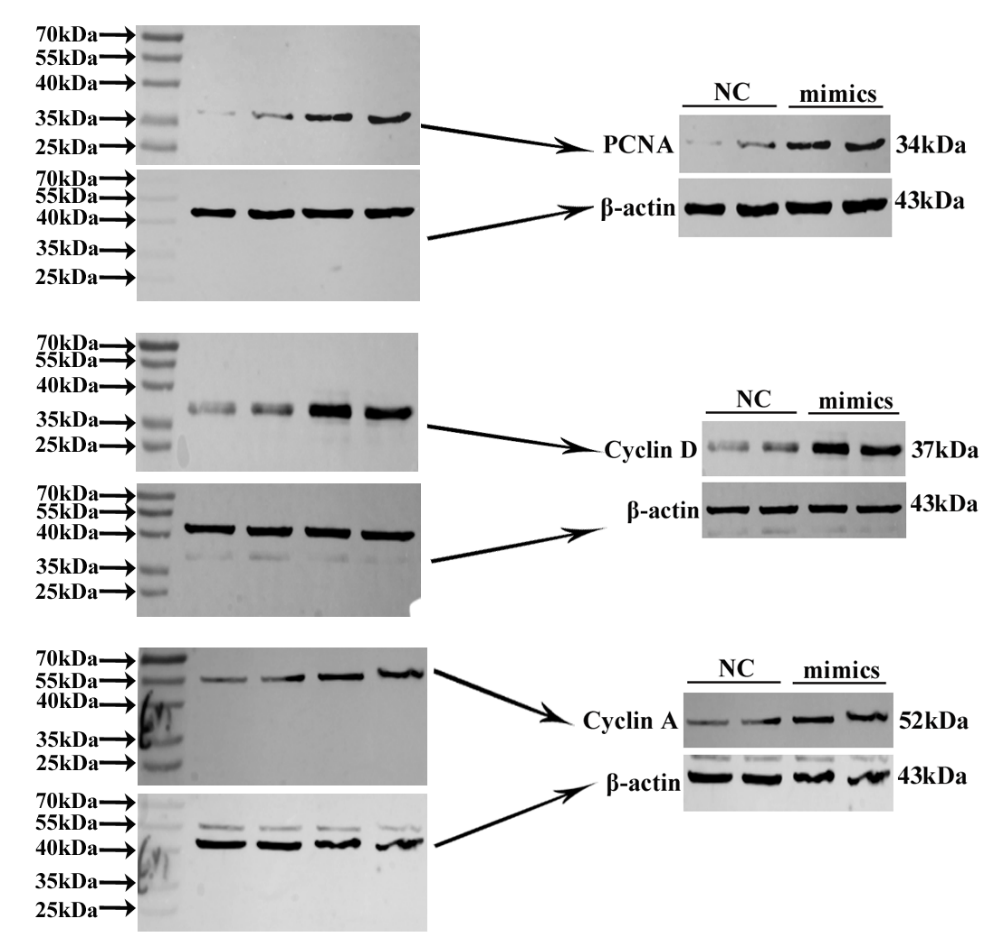


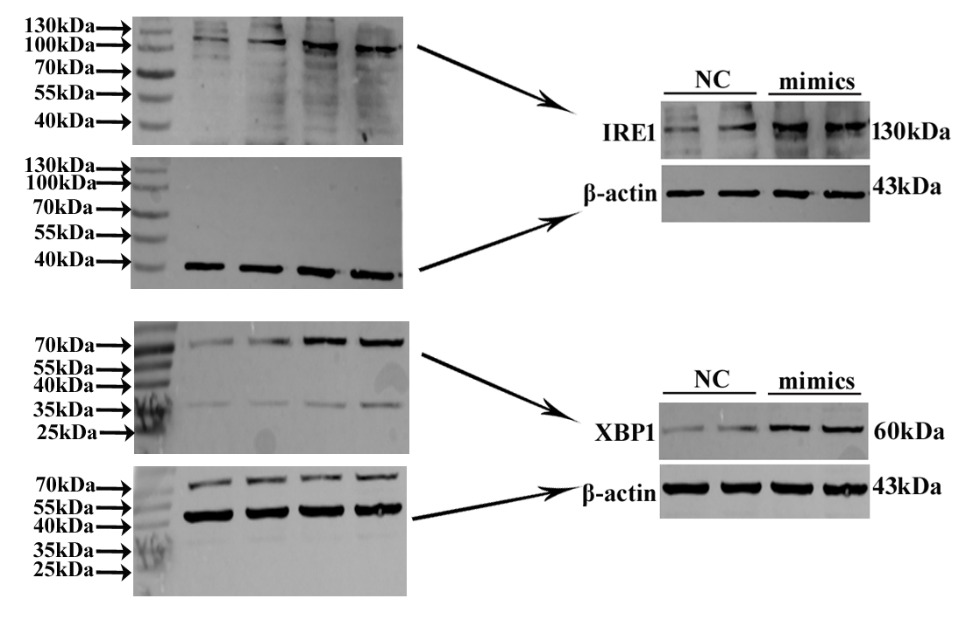


Figure S3


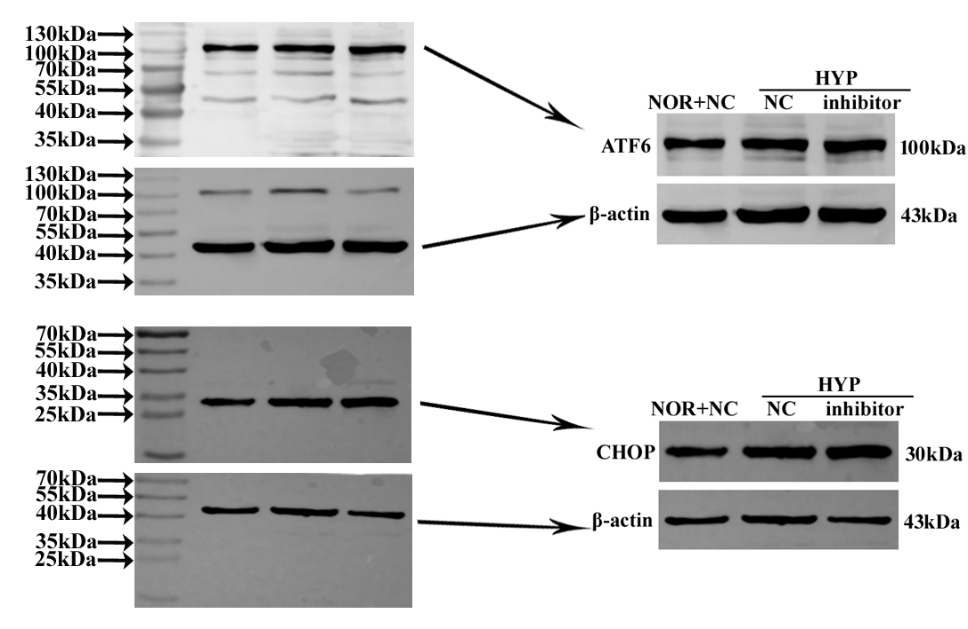


Figure S4


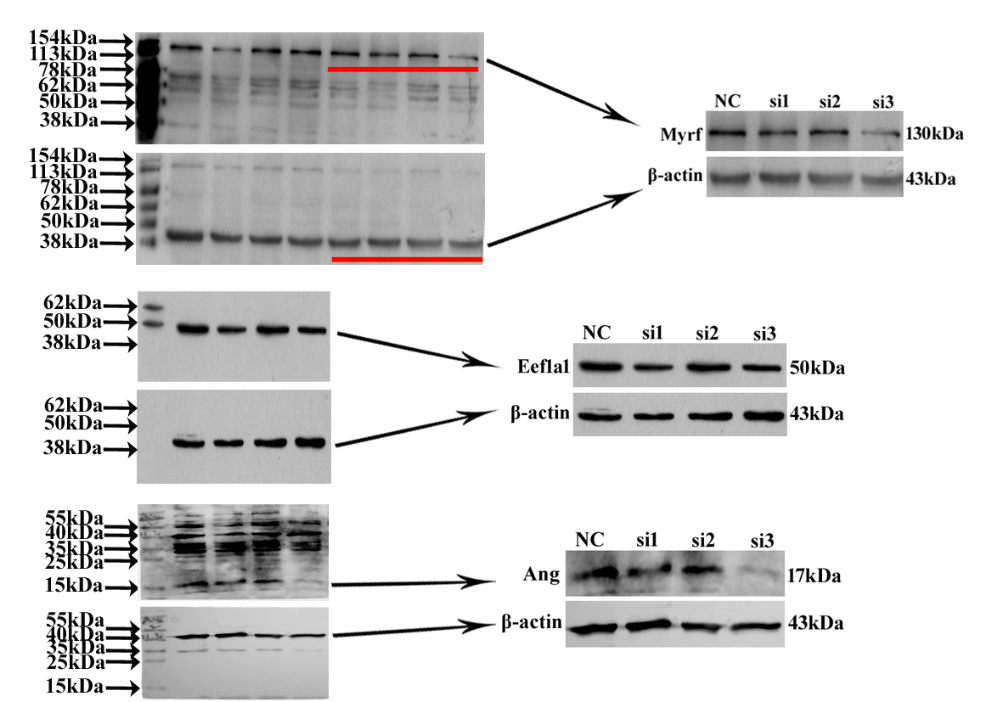

Supplement: Supplementary file 2 — Data S1: cpr70238‐sup‐0002‐Supinfo02.docx. [file CPR-9999-e70238-s002.docx]
